# Prolonged shedding of SARS-CoV-2 at high viral loads amongst hospitalised immunocompromised persons living with HIV, South Africa

**DOI:** 10.1093/cid/ciac077

**Published:** 2022-02-02

**Authors:** Susan Meiring, Stefano Tempia, Jinal N Bhiman, Amelia Buys, Jackie Kleynhans, Mvuyo Makhasi, Meredith McMorrow, Jocelyn Moyes, Vanessa Quan, Sibongile Walaza, Mignon du Plessis, Nicole Wolter, Anne von Gottberg, Cheryl Cohen, COVID-19 shedding study group

**Affiliations:** Division of Public Health Surveillance and Response, National Institute for Communicable Diseases, a Division of the National Health Laboratory Service, Johannesburg, South Africa; School of Public Health, Faculty of Health Sciences, University of the Witwatersrand, Johannesburg, South Africa; Centre for Respiratory Diseases and Meningitis, National Institute for Communicable Diseases, a Division of the National Health Laboratory Service, Johannesburg, South Africa; Influenza Division, Centers for Disease Control and Prevention, Atlanta, Georgia, United States of America; School of Public Health, Faculty of Health Sciences, University of the Witwatersrand, Johannesburg, South Africa; Centre for Respiratory Diseases and Meningitis, National Institute for Communicable Diseases, a Division of the National Health Laboratory Service, Johannesburg, South Africa; School of Pathology, Faculty of Health Sciences, University of the Witwatersrand, Johannesburg, South Africa; Centre for Respiratory Diseases and Meningitis, National Institute for Communicable Diseases, a Division of the National Health Laboratory Service, Johannesburg, South Africa; Centre for Respiratory Diseases and Meningitis, National Institute for Communicable Diseases, a Division of the National Health Laboratory Service, Johannesburg, South Africa; School of Public Health, Faculty of Health Sciences, University of the Witwatersrand, Johannesburg, South Africa; Centre for Respiratory Diseases and Meningitis, National Institute for Communicable Diseases, a Division of the National Health Laboratory Service, Johannesburg, South Africa; Influenza Division, Centers for Disease Control and Prevention, Atlanta, Georgia, United States of America; Division of Viral Diseases, US Centers for Disease Control and Prevention, Atlanta, Georgia, United States of America; Centre for Respiratory Diseases and Meningitis, National Institute for Communicable Diseases, a Division of the National Health Laboratory Service, Johannesburg, South Africa; Division of Public Health Surveillance and Response, National Institute for Communicable Diseases, a Division of the National Health Laboratory Service, Johannesburg, South Africa; Centre for Respiratory Diseases and Meningitis, National Institute for Communicable Diseases, a Division of the National Health Laboratory Service, Johannesburg, South Africa; School of Public Health, Faculty of Health Sciences, University of the Witwatersrand, Johannesburg, South Africa; Centre for Respiratory Diseases and Meningitis, National Institute for Communicable Diseases, a Division of the National Health Laboratory Service, Johannesburg, South Africa; School of Pathology, Faculty of Health Sciences, University of the Witwatersrand, Johannesburg, South Africa; Centre for Respiratory Diseases and Meningitis, National Institute for Communicable Diseases, a Division of the National Health Laboratory Service, Johannesburg, South Africa; School of Pathology, Faculty of Health Sciences, University of the Witwatersrand, Johannesburg, South Africa; Centre for Respiratory Diseases and Meningitis, National Institute for Communicable Diseases, a Division of the National Health Laboratory Service, Johannesburg, South Africa; School of Pathology, Faculty of Health Sciences, University of the Witwatersrand, Johannesburg, South Africa; Centre for Respiratory Diseases and Meningitis, National Institute for Communicable Diseases, a Division of the National Health Laboratory Service, Johannesburg, South Africa; School of Public Health, Faculty of Health Sciences, University of the Witwatersrand, Johannesburg, South Africa

**Keywords:** COVID-19, respiratory virus, shedding duration, HIV, immunocompromised

## Abstract

**Background:**

We assessed SARS-CoV-2 RNA shedding duration and magnitude amongst persons living with HIV (PLHIV).

**Methods:**

From May through December 2020, we conducted a prospective cohort study at 20 hospitals in South Africa. Adults hospitalised with symptomatic COVID-19 were enrolled and followed every two days with nasopharyngeal/oropharyngeal (NP/OP) swabs until documentation of cessation of SARS-CoV-2 shedding (two consecutive negative NP/OP swabs). Real-time reverse transcription-polymerase chain reaction testing for SARS-CoV-2 was performed and Cycle-threshold (C_t_) values <30 were considered a proxy for high SARS-CoV-2 viral load. Factors associated with prolonged shedding were assessed using accelerated time-failure Weibull regression models.

**Results:**

Of 2,175 COVID-19 patients screened, 300 were enrolled and 257 individuals (155 HIV-uninfected and 102 PLHIV) had >1 swabbing visit (median 5 visits (range2-21)). Median time to cessation of shedding was 13 days (inter-quartile range (IQR)6-25) and did not differ significantly by HIV-infection.

**Discussion:**

Amongst a subset of 94 patients (41 PLHIV and 53 HIV-uninfected) with initial respiratory sample C_t_-value <30, median time of shedding at high SARS-CoV-2 viral load was 8 days (IQR4-17). This was significantly longer in PLHIV with CD4 count<200cells/µl, compared to HIV-uninfected persons (median 27 days (IQR8-43) versus 7 days (IQR 4-13); aHR 0.14, 95%CI 0.07-0.28, p<0.001), with similar results in unsuppressed-HIV versus HIV-uninfected persons.

**Conclusion:**

Although SARS-CoV-2 shedding duration did not differ significantly by HIV-infection, amongst a subset with high initial SARS-CoV-2 viral loads, immunocompromised PLHIV shed SARS-CoV-2 at high viral loads for longer than HIV-uninfected persons. Better HIV control may potentially decrease transmission time of SARS-CoV-2.

*Conclusion*

Although SARS-CoV-2 shedding duration did not differ significantly by HIV-infection, amongst a subset with high initial SARS-CoV-2 viral loads, immunocompromised PLHIV shed SARS-CoV-2 at high viral loads for longer than HIV-uninfected persons. Better HIV control may potentially decrease transmission time of SARS-CoV-2.

### Keywords

COVID-19; respiratory virus; shedding duration; HIV; immunocompromised

### Introduction

Immunocompromised persons are thought to shed SARS-CoV-2 for a longer duration, increasing time for viral transmission and potentially driving within host viral evolution.[1–3] South Africa has 7,500,000 persons living with HIV (PLHIV) and there are no systematically collected data on duration of SARS-CoV-2 shedding in PLHIV. We hypothesised that PLHIV may shed SARS-CoV-2 for a longer period of time and at a higher viral load than HIV-uninfected individuals.

SARS-CoV-2 shedding from the upper respiratory tract extends for a mean of 17 days (15.5-18.6).[4] Unlike SARS-CoV-1, viral shedding of SARS-CoV-2 from the upper respiratory tract peaks on or before symptom onset, allowing some viral transmission to occur before symptom onset in infected individuals.[4–8] Quantifying SARS-CoV-2 viral load ($Log_{10}$ RNA copies/mL) is calculated by converting qualitative rRT-PCR cycle threshold values using calibration curves based on quantified E-gene in vitro RNA transcripts.[9] However, studies on SARS-CoV-2 infectiousness indicate that successful virus isolation is most likely from specimens with a real-time RT-PCR cycle threshold ($C_t$) value<34(~7 $Log_{10}$ copies/mL), however not all these specimens may be positive for viable virus. [9,10] Using this threshold, infectiousness declines significantly 8 days after becoming symptomatic, even though SARS-CoV-2 viral RNA persistence has been shown to occur for months in some individuals.[9,11,12]

Various factors have been associated with increased shedding duration, including increased age, male sex, severity of illness and use of corticosteroids.[13,14] There are several published case reports indicating prolonged SARS-CoV-2 transmission in immunocompromised persons, mostly with cancers or autoimmune conditions, and more recently in an HIV-infected immunocompromised person.[15–18]

We aimed to evaluate the overall duration of SARS-CoV-2 shedding in upper respiratory tract specimens and duration of high viral load shedding in a cohort of PLHIV and HIV-uninfected persons hospitalised with COVID-19 in South Africa. We also estimated SARS-CoV-2 shedding duration in stool and blood specimens, and described serologic responses to SARS-CoV-2 infection.

## Methods

*Study design and setting*

We conducted a prospective cohort study from May 1 through December 31, 2020 (spanning the first wave and beginning of the second wave of the COVID-19 pandemic in South Africa). Persons hospitalised for symptomatic COVID-19 at one of 20 hospitals situated in 8 of the 9 South African provinces were invited to participate in the study if they met the following inclusion criteria: aged 18 years and older, laboratory-confirmed diagnosis of SARS-CoV-2 in the previous 5 days, resided within a 50km radius of the hospital, and had laboratory-confirmation of their HIV status.

Using standardised case report forms, demographic and clinical details were collected at enrolment, daily whilst in hospital, and at discharge from hospital/cessation of shedding/death of the participant. Included variables are detailed in supplementary text.

At enrolment and every second day thereafter, until cessation of shedding or death, a combined nasopharyngeal/oropharyngeal (NP/OP) swab was collected by trained nursing staff, using two flocked nylon plastic shaft swabs (one for the nasopharynx and one for the oropharynx) which were then inserted together into Universal Transport Medium and transported on ice to the laboratory. Sensitivity of the combined NP/OP swab has been shown to be 97% for detection of SARS-CoV-2 in the upper airways.[19,20] A rectal swab or stool was collected from patients at the same time intervals and transported to the laboratory in sealed containers. Results available in supplementary text. Patients were followed up at home if they were still SARS-CoV-2 positive on NP/OP swab on discharge from hospital.

Whole blood and serum specimens were taken on enrolment and at days 7, 14 and 21 post symptom-onset for SARS-CoV-2 testing and serology. All specimens were transported to the National Institute for Communicable Diseases (NICD) in Johannesburg for processing and testing.

*Laboratory diagnostics*

Real-time reverse transcription polymerase chain reaction (rRT-PCR) for the qualitative detection of nucleic acid from SARS-CoV-2 was performed on NP/OP, stool/rectal swabs and blood specimens using the Allplex™ nCoV 2019 kit (Seegene, Seoul, South Korea). Specimens were considered positive for SARS-CoV-2 nucleic acids if the $C_t$ was <40 for ≥1 of 3 gene targets. A nucleocapsid gene (N gene) $C_t$-value <30 on NP/OP specimens was used as a proxy for a high viral load based on published data showing a high correlation between low $C_t$-values (using various gene targets), high viral load and increased odds of shedding cultivable virus.[10,12,21,22]

Antibody detection against trimeric ectodomain HexaPro spike protein was performed on serum specimens as described previously.[23,24] Absorbance at 450 nm was measured and specimens with Optical Density (OD)>0.4 were considered positive for anti-spike protein antibodies.

SARS-CoV-2 sequencing was performed on the first NP/OP specimens on enrolment from ten randomly selected participants of 29, who demonstrated high viral load shedding for >14 days. Clade and lineage assignments were made using the online Nextclade (https://clades.nextstrain.org/) and Pangolin (https://pangolin.cog-uk.io/) applications, which also enable identification of known variants of concern as well as novel mutations. See supplementary methods for more details.

*Definitions*

Shedding was defined as presence of SARS-CoV-2 nucleic acid in a specimen as detected by a positive SARS-CoV-2 rRT-PCR result. Participants were deemed to have stopped shedding SARS-CoV-2 from the respiratory tract once two consecutive NP/OP swabs, taken at least two days apart, tested negative for all 3 gene targets on rRT-PCR. Time to cessation of shedding was taken from date of symptom onset to the date of the last rRT-PCR SARS-CoV-2 positive NP/OP swab prior to the two consecutive negative swabs. Participants were deemed to be shedding SARS-CoV-2 virus at high viral loads if their NP/OP swab N gene $C_t$-value was <30. Persistence of high viral load was measured in the subgroup of individuals whose first study NP/OP swab had an N gene $C_t$-value <30 and was measured from date of symptom onset to the last date where NP/OP swab N gene $C_t$-value was <30. PLHIV were deemed to be significantly immunocompromised if their CD4 T-lymphocyte count was <200 cells/µl, and not HIV virally suppressed if their HIV viral load measured ≥400 copies/ml in the 3 months before hospital admission.[25,26] Severity of COVID-19 was categorised using respiratory rate at time of admission according to the WHO clinical classification of COVID-19 and a quick Sequential (Sepsis-related) Organ Failure Assessment (qSOFA) score.[27,28]

*Statistical analysis*

Data were captured on a real-time data capture (REDCAP) database and transferred onto a password protected Microsoft Access database management system.[29] Statistical analysis was performed using Stata version 14 (StataCorp Inc., College Station, Texas, USA).

We used Kaplan Meier estimates and Weibull accelerated failure time regression models to measure time from symptom onset to i) cessation of shedding and ii) low viral load shedding (at $C_t$-values ≥ 30). The Weibull model was chosen as it allows explanatory variables to proportionately increase or decrease the time to the end-point.[30] Hazard ratios measured the hazard of reaching the end-points with a ratio of <1 indicating a longer duration of shedding or a longer duration of high viral load shedding. P-values <0.2 on univariate analysis were included in all multivariable models and non-significant variables (p-value>0.05) were dropped using step-wise manual backward elimination. Patients with missing data were dropped from the models.

*Ethics*

Ethics clearance for the study was obtained through various university health research ethics committees (HREC), namely University of the Witwatersrand HREC (Medical) (M160667); Stellenbosch University HREC (15206); University of Pretoria HREC (256/2020) and University of the

Free State HREC (HSD2020/0625). Permission to conduct the study was obtained from each Provincial Department of Health Research Committee. All participants gave written informed consent to participate in the study.

### Results

*Participant inclusion and characterisation*

From May 1 through December 31, 2020, 2175 hospitalised COVID-19 patients were screened for enrolment at 20 sentinel hospitals across South Africa (Figure 1). Of the 1875 not enrolled: 225 refused consent, 186 were transferred out of the hospital on the day of screening, 64 were minors, and 1400 did not meet the eligibility criteria (Figure 1 and Supplementary Table 1). Three hundred persons were enrolled into the study of which 257 had at least 2 study visits (range2-21), with a median of 5 visits per participant (Supplementary Figure 1). Of these, 155 persons were HIV-uninfected and 102 PLHIV (Figure 1).

Of the 257 patients included in the cohort: median age was 46 years (interquartile range (IQR)35-58 years), PLHIV were significantly younger than HIV-uninfected persons (median of 41 years in PLHIV and 49 years in HIV-uninfected, $p<0.001$). Sixty-one percent of participants were female with significantly more females than males represented amongst PLHIV ($p<0.001$). The majority of participants (86%) were of black African descent. Overall, 33% had underlying hypertension (40% HIV-uninfected vs 24%PLHIV, $p=0.01$), 21% had diabetes (28% HIV-uninfected vs 11%PLHIV, $p=0.002$), 16% were obese (19% HIV-uninfected vs 13%PLHIV, $p=0.25$), and 8% had underlying tuberculosis (2% HIV-uninfected vs 16%PLHIV, $p<0.001$). Thirteen percent were current cigarette smokers (Table 1). Of PLHIV, 84% were currently receiving antiretroviral therapy, median CD4 T-cell count was 221 cells/µl (IQR48-443) (with 53% having CD4 T-cell count≥200cells/µl), and 59% were HIV virally suppressed (HIV viral load<400 copies/ml).

Patients were admitted for a median of 9 days (IQR6-13) in hospital. Eight participants had unknown outcome. Overall, 10% (24/250) of participants died, 8% (19) during their hospital admission and a further 2% (5) within 2months post-discharge. This did not differ significantly by HIV-status. Of 214 participants with known parameters to assess disease severity, most (64%, 137/214) had moderate disease severity, with 18% (39/214) having mild and 18% (38/214) having severe COVID-19. HIV-uninfected persons and PLHIV had similar clinical presentation. The most common presenting symptoms were cough (79%, 192/243), dyspnoea (66%, 160/243), chest pain (63%, 153/243), malaise (52%, 127/243), and fever (45%, 111/243). Oxygen therapy was required for 53% (125/235), non-invasive ventilation for 7% (15/227), and invasive ventilation for 5% (11/230). Ten percent (23/235) were admitted to an intensive care unit. Glucocorticoids were given to 34% (84/250) of individuals whilst in hospital (Table 1).

*Factors associated with duration of shedding*

Of the 257 participants with at least 2 swabbing visits: 186 were followed up until cessation of SARS-CoV-2 shedding, 14 died prior to stopping shedding, and 57 were lost to follow up. Overall there were 4881 patient days of observation. The median duration of SARS-CoV-2 shedding from the 257 participants was 13 days (IQR6-25) from symptom onset: 15 days (IQR8-27) in HIV-uninfected versus 11 days (IQR5-22) in PLHIV (adjusted hazard ratio (aHR) 1.16, 95%CI 0.8-1.68, p=0.44). Duration of SARS-CoV-2 shedding was not significantly different by different age categories, sex, HIV immunosuppression, severity of illness, and use of glucocorticoids. However, after adjusting for confounders on multivariable analysis, the duration of SARS-CoV-2 detection was 45% longer in obese compared to non-obese persons (18 days (IQR11-34) vs 13 days (IQR6-25), aHR 0.55, 95%CI 0.33-0.90, p=0.02) (Table 2; Figure 2; Supplementary Table 2).

*Factors associated with shedding potentially infectious virus*

Of a subset of 94 participants with N gene $C_t$-value<30 at enrolment (41 PLHIV and 53 HIV-uninfected persons), the median duration of SARS-CoV-2 shedding at N gene $C_t$-value<30 from symptom onset was 8 days (IQR4-17). On multivariable analysis, when adjusting for age and glucocorticoid use, PLHIV with a CD4 cell count<200 cells/µl shed at high SARS-CoV-2 viral loads for longer (median 27days, IQR8-43, aHR 0.14, 95%CI 0.07-0.28, p<0.001), whereas PLHIV with CD4 count≥200 cells/µl shed at high SARS-CoV-2 viral loads for a similar time period (median 7days, IQR4-10, aHR 1.14, 95%CI 0.56-2.31, p=0.71), compared to HIV-uninfected persons (median 7days, IQR4-13). Similarly, PLHIV with HIV viral loads≥400 copies/ml were more likely to shed SARS-CoV-2 at high viral loads for longer (26days, IQR10-41, unadjusted HR 0.34, 95%CI 0.18-0.64, p<0.001) than PLHIV with HIV viral suppression (6days, IQR4-8, unadjusted HR 1.77, 95%CI 0.88-3.56, p=0.107) and those who were HIV-uninfected (7days, IQR4-13) (Table 3; Figure 3; Supplementary Table 3).

*SARS-CoV-2 antibodies*

The proportion of participants with positive anti-spike protein antibodies increased from 80% (161/201) by day 7 to 87% (135/156) at day 14 post-symptom onset. PLHIV with CD4 count<200 cells/µl were significantly less likely to develop anti-spike protein antibody titres by day 7, 14 or 21 post symptom-onset than HIV-uninfected persons (Table 4). Median anti-spike protein antibody titres were significantly higher by 7 and 14days post-symptom onset in HIV-uninfected persons (Figure 4). By day 21, there were 4 HIV-uninfected and 10 PLHIV (8 with CD4 counts<200cells/µl, one with CD4 count>200cells/µl and one with unknown CD4 count) who did not develop anti-spike protein antibodies. Seven (2 HIV-uninfected and 5 PLHIV with CD4 count<200cells/µl) of these 14 shed SARS-CoV-2 viral RNA from the upper respiratory tract for 20days or longer post symptom-onset (all 5 PLHIV still had N gene $C_t$-values<26 by day20).

*SARS-CoV-2 sequencing of upper respiratory specimens on enrolment*

Eight different SARS-CoV-2 phylogenetic lineages were detected from enrolment NP/OP swabs from the ten randomly selected participants demonstrating high SARS-CoV-2 viral load shedding for >2 weeks. These included two participants with B.1, two B.1.140, and one each of B.1.1.57, B.1.1.448, B.1.1.382, C.1, C.2, and C.6 lineages.

### Discussion

In this South African cohort of 257 persons (including 102 PLHIV) hospitalised with COVID-19, the median duration of SARS-CoV-2 shedding was 13 days and did not differ significantly by HIV status. Amongst a subset with high initial SARS-CoV-2 viral shedding, duration of high viral load SARS-CoV-2 shedding (at N gene $C_t$-value<30) was 8 days overall, and significantly prolonged in PLHIV with HIV immunosuppression and/or high HIV viral load. SARS-CoV-2 nucleic acids were detected in blood and stool/rectal swab specimens at low RNA concentrations in both PLHIV and HIV-uninfected persons. Immunocompromised PLHIV were also less likely to develop anti-spike protein antibody by day7 or 14 compared to HIV-uninfected persons. A wide variety of SARS-CoV-2 phylogenetic lineages were detected in the small group of participants who shed SARS-CoV-2 at high viral loads for more than 2 weeks.

We showed prolonged duration of high viral load SARS-CoV-2 shedding in PLHIV with low CD4 cell counts. Prolonged influenza virus shedding has been described in immunocompromised PLHIV and it has been associated with viral evolution and antigenic drift within individual immunocompromised patients, similar to that occurring in communities.[31–34] Further studies are needed to explore SARS-CoV-2 intra-individual adaptations in a similar manner to influenza, particularly in PLHIV.

The national prevalence of HIV infection in South Africa is 13%, and it is estimated that 4.4 million of the 7.5 million PLHIV in South Africa are not HIV virally suppressed. (UNAIDS https://www.unaids.org/en/regionscountries/countries/southafrica). If immunocompromised PLHIV shed SARS-CoV-2 for longer at higher SARS-CoV-2 viral loads, this could potentially impact the risk of viral evolution and transmission, especially in South Africa. Alternately, PLHIV with CD4 counts of ≥200cells/µl shed for a similar time and at a similar SARS-CoV-2 viral load than HIV-uninfected persons. This finding highlights the importance of early HIV diagnosis and treatment with the goal of HIV viral suppression, thus improving the health of PLHIV and minimising transmission risk of SARS-

CoV-2 in the general population. The study was conducted prior to any COVID-19 vaccinations being available in South Africa. Now that vaccines are available, prioritising COVID-19 vaccination of PLHIV may be another strategy for minimising SARS-CoV-2 spread.

Recently, accelerated SARS-CoV-2 viral evolution was described in an immunocompromised host with persistent SARS-CoV-2 infection, as well as in a HIV-infected host with poor HIV viral suppression.[17,35] SARS-CoV-2 viral mutations occur commonly within hosts and within populations and prolonged high viral load shedding provides a good opportunity for virus evolution and transmission of potential viral mutations. Between June and October 2020, 44 different SARS-CoV-2 lineages were detected in South Africa, with the B.1, B.1.1, B.1.1.448, B.1.1.54, and C.1 lineages accounting for 59% of all sequences (n=1705).[36] In this study the multiple SARS-CoV-2 phylogenetic lineages detected reflect the lineage diversity during the earlier waves in South Africa, indicating that persistent shedding was not dominated by a specific lineage or variant. All lineages detected in these individuals had been identified in national genomic surveillance efforts, with 7 of the 8 being lineages first detected in South Africa.[36] Going forward we aim to investigate within host viral evolution by sequencing sequential specimens of the participants with prolonged high viral load shedding, many of whom were immunocompromised PLHIV.

Obese patients in our study showed a 45% increase in median time of SARS-CoV-2 shedding compared to their non-obese counterparts. However, obesity was not associated with prolonged duration of high viral load SARS-CoV-2 shedding. Recent studies indicate that obesity may be associated with prolonged SARS-CoV-2 shedding, in a similar manner to prolonged shedding seen in obese adults with influenza A virus. [37,38] See supplementary material for further discussion.

Most participants developed anti-spike protein antibodies by day 14, with fewer immunocompromised PLHIV developing antibodies compared to HIV-uninfected persons. Overall lower median anti-spike protein antibody titres were detected in PLHIV. Our study showed 14 individuals (4 HIV-uninfected persons and 10 PLHIV - 8 of whom had low CD4 count) who did not develop antibodies within the initial 21 days' follow-up period, half of which showed prolonged shedding (including 5 PLHIV with CD4 count<200 cells/µl). Other studies have found similar findings in immunocompromised individuals with prolonged viral shedding having negative seroconversion.[15,16]

The cohort of participants from 20 public hospitals is likely representative of the hospitalised COVID-19 South African adult population, however the analysis did not account for within-hospital correlations due to case mix, treatment strategies or inter-hospital differences in quality of care.[39] Thrice weekly collection of NP/OP swabs to detect ongoing SARS-CoV-2 viral shedding may result in underestimated viral shedding due to interval censoring of the data. A major limitation of our study is the use of qualitative $C_t$-values as a proxy for viral load. Although all specimens were tested in the same laboratory, using standardised technique and assays, quantitative RT-PCR was not performed using calibration curves to determine viral load. In addition, we were unable to perform viral culture to confirm shedding of infectious virus at proxy $C_t$-values<30. Our findings may not be generalizable to outpatients with mild COVID-19, as hospitalised COVID-19 patients may represent more severe disease and therefore shed for a longer duration. Non-enrolment of participants due to unavailability of HIV test data (37% (702/1875) of non-enrolments) may have introduced bias due to potential differences in persons willing to undergo HIV testing versus those who are unwilling. Previous South African studies have shown similar unknown HIV results, as opt-out policies on HIV testing are not routinely followed.[39] Although sensitivity analyses in our study did not show much effect when excluding patients who died (data not shown), it should be noted that in-hospital mortality in our study was low (8%) compared to 23% in Jassat, et al., as patients too ill to consent would have been excluded.[39] Follow-up of participants' post-hospital discharge was challenging due to stigma of COVID-19 in the communities, safety concerns and travel limitations. Fifty-eight participants were lost to follow up prior to cessation of shedding, however the accelerated time failure Weibull regression analysis included patient data up until the patient was lost to follow-up, thereby utilising all data generated.

Immunocompromised persons living with HIV shed SARS-CoV-2 at a higher viral load for a longer duration, and have lower anti-spike protein antibody levels than HIV-uninfected persons, which could lead to increased transmission and ongoing viral evolution of SARS-CoV-2, unless HIV control is achieved. During the COVID-19 pandemic, access to HIV testing and treatment with an aim for HIV viral suppression remains extremely important. In high HIV-prevalence settings better HIV control may potentially decrease the transmission period of SARS-CoV-2 in communities.

NOTES

### Acknowledgement

The authors would like to thank the participants in the study for their time and patience, as well as the GERMS-SA surveillance officers and team members of the Centre for Respiratory Diseases and Meningitis at the NICD.

Although not part of the COVID-19 Shedding Study Group, the following individuals assisted in the study:

Livingstone Hospital: Vanessa Pearce, Microbiology, National Health Laboratory Service, Eastern Cape; Pelonomi Academic Hospital: Dewald Steyn, Internal Medicine, Pelonomi Hospital, Bloemfontein, Free State; Dr George Mukhari Academic Hospital: Temitayo Famoroti, Clinical Virology, National Health Laboratory Service, Gauteng; and Nkosinathi Mdladla, Medicine, Dr George Mukhari Hospital, Gauteng; King Edward III Hospital: Prasha Mahabeer, Microbiology, National Health Laboratory Service, Kwazulu-Natal; Mankweng Hospital: Samuel Cana, Medicine, Mankweng Hospital, Limpopo.

### Members of the COVID-19 Shedding Study Group and their affiliations

Members from the various hospital sites that made up the COVID-19 Shedding Study Group.

**Dora Nginza Provincial and Livingstone Hospitals:**

John Black:  Department of Medicine, Walter Sisulu University, Port Elizabeth, Eastern Cape

**Pelonomi Academic and Universitas Academic Hospitals:**

Dominique Goedhals: Division of Virology, National Health Laboratory Service (NHLS)/ University of the Free State, Bloemfontein, Free State

Bonnie Maloba: Microbiology, University of the Free State, Bloemfontein Free State

Samantha Potgieter: Internal Medicine, Division Infectious Diseases, University of the Free State, Bloemfontein, Free State

**Charlotte Maxeke Johannesburg Academic Hospital:**

Marianne Black: Department of Clinical Microbiology and infectious Diseases, University of the Witwatersrand, Johannesburg, Gauteng Microbiology, NHLS, Johannesburg, Gauteng

Vindana Chibabhai: Clinical Microbiology, NHLS, Johannesburg, Gauteng Clinical Microbiology and Infectious Diseases, University of the Witwatersrand, Johannesburg, Gauteng

Nonhlanhla Mbenenge: Virology, NHLS, Johannesburg, Gauteng Virology, University of the Witwatersrand, Johannesburg Gauteng

Trusha Nana: Microbiology, NHLS, Johannesburg, Gauteng Department of Clinical Microbiology and Infectious Diseases, University of Witwatersrand, Johannesburg, Gauteng

Sarah Stacey: Department of Internal Medicine, University of the Witwatersrand, Johannesburg, Gauteng Department of Internal Medicine, Charlotte Maxeke Johannesburg Academic Hospital, Johannesburg, Gauteng

Florette Treurnicht: Virology, NHLS, Johannesburg, Gauteng

**Dr George Mukhari Academic Hospital**

Masego Moncho: Microbiology, NHLS, Pretoria, Gauteng Microbiology, Sefako Makgatho Health Sciences University, Pretoria, Gauteng

Maphoshane Nchabeleng: Microbiology, NHLS, Pretoria, Gauteng Microbiology, Sefako Makgatho Health Sciences University, Pretoria, Gauteng

Grace Shikwambane-Ntlemo: Microbiology, NHLS, Pretoria, Gauteng Microbiology, Sefako Makgatho Health Sciences University, Pretoria, Gauteng Helen Joseph Hospital

Rispah Chomba: Clinical microbiology, NHLS, Johannesburg, Gauteng Department of clinical microbiology and infectious diseases, School of Pathology, University of the Witwatersrand, Johannesburg, Gauteng

Jeremy Nel: Division of Infectious Diseases, Department of Medicine, University of the Witwatersrand, Johannesburg, Gauteng

**Steve Biko Academic Hospital**

Anwar Hoosen: Medical Microbiology, University of Pretoria, Pretoria, Gauteng Microbiology Laboratory, NHLS, Tshwane Academic Division, Pretoria, Gauteng

Mohamed Said: Medical Microbiology, University of Pretoria, Pretoria, Gauteng Tshwane Academic Division, NHLS, Pretoria, Gauteng

**Addington Hospital**

Junaid Bayat: Medicine, Addington Hospital, Durban, KwaZulu Natal

Lisha Sookan: Medical Microbiology, NHLS, Durban, KwaZulu Natal Medical Microbiology, University of KwaZulu Natal, Durban, KwaZulu Natal

Khine Swe Swe Han: Medical Microbiology, NHLS, Durban, KwaZulu Natal

**Edendale and Northdale Hospitals**

Surendra Sirkar: Department of Health, Northdale Hospital, Pietermaritzburg, KwaZulu Natal

**Greys Hospital**

Halima Dawood: Medicine, Greys hospital, Pietermaritzburg, KwaZulu Natal Caprisa, University of Kwazulu Natal, Durban, KwaZulu Natal

Sumayya Haffejee: Medical Microbiology, NHLS, Pietermaritzburg, KwaZulu Natal Medical Microbiology, University of KwaZulu-Natal, Pietermaritzburg, KwaZulu Natal

**King Edward III Hospital**

Somasundram Pillay: Internal Medicine, University of KwaZulu-Natal, Durban, KwaZulu Natal Internal Medicine, King Edward VIII Hospital, Durban, KwaZulu Natal

Praksha Ramjathan: Medical Microbiology, NHLS, Durban, KwaZulu Natal Medical Microbiology, University of KwaZulu Natal, Durban, KwaZulu Natal

**RK Khan Hospital**

Nomonde Mvelase: Medical Microbiology, NHLS, Durban, KwaZulu Natal Medical Microbiology, University of KwaZulu-Natal, Durban, KwaZulu Natal

Javid Mulla: Medicine, RK Khan and King Edward III Hospitals, Durban, KwaZulu Natal

**Polokwane Provincial and Mankweng Hospitals**

Ruth Lekalakala-Mokaba: Pathology , University of Limpopo, Polokwane , Limpopo Medical Microbiology, NHLS, Polokwane, Limpopo

**Rob Ferreira Hospital**

Matamela Madua: Medicine, Rob Ferreira Hospital, Nelspruit, Mpumalanga

Sindile Ntuli : Medical Microbiology, NHLS, Nelspruit, Mpumalanga Tshepong Hospital

Ebrahim Variava: Medicine, Klerksdorp/Tshepong Hospital Complex, Klerksdorp, North West

**Mitchells Plain Hospital**

Thomas Crede: Medicine, Mitchells Plain Hospital, Cape Town, Western Cape Medicine, University of Cape Town, Cape Town, Western Cape

**Tygerberg Hospital**

Kessendri Reddy: Division of Medical Microbiology and Immunology, Department of Pathology, Stellenbosch University, Cape Town, Western Cape Medical Microbiology, NHLS, Tygerberg Hospital, Cape Town, Western Cape

Jantjie Taljaard: Division of Infectious Diseases, Department of Medicine, Tygerberg Hospital and Stellenbosch University, Cape Town, Western Cape

Andrew Whitelaw: Division of Medical Microbiology and Immunology, Department of Pathology, Stellenbosch University, Cape Town, Western Cape Medical Microbiology, NHLS, Tygerberg Hospital, Cape Town, Western Cape

### Author contributions

Conception and design of study: All authors

Data collection and laboratory processing: SM, ST, JNB, AB, JK, MM, MMcM, JM, VQ, SW, MdP, NW, AvG, CC

Analysis and interpretation: SM, ST, CC, AvG, JNB, AB, JK, MMcM, JM, VQ, SW

Drafting or critical review of the article: All authors

### Disclaimer:

The findings and conclusions in this report are those of the authors and do not necessarily represent the official position of the Centers for Disease Control and Prevention.

### Funding statement

The study was funded by the Wellcome Trust [grant number 221003/Z/20/Z] in collaboration with the Foreign, Commonwealth and Development Office, United Kingdom; the U.S. Centers for Disease

Control and Prevention [co-operative agreement number: 6 U01IP001048-04-02]; as well as the National Institute for Communicable Diseases, a division of the National Health Laboratory Service, South Africa. The funding agencies had no role in the development of the study protocol, data collection, analysis and interpretation, writing of the report, or decision to submit.

### Conflicts of interest

Susan Meiring reports a grant from Sanofi Pasteur for research outside the submitted work. Anne von Gottberg reports grants from US CDC Atlanta and CDC SA, PATH, Wellcome Trust, Sanofi, and from South African MRC, European Union, Jack Ma Fndt. (Allibaba), EDCTP partnership, BMFG through ASLM and Africa CDC, Fleming Fund Regional Africa WGS (SEQAFRICA), outside the submitted work. Cheryl Cohen reports grants from Wellcome Trust, SA Medical Research Council (SAMRC), and US CDC related to the summited work and grant to institute for COVID-19 Research from Sanofi Pasteur outside of the submitted work; participation on a Member Scientific Advisory Committee – BCHW: Burden of COVID-19 among health care workers, assessing infection, risk factors, vaccine effectiveness, working experiences and one-health implications: a mixed methodology, multisite international study, study is focused on COVID-19 – no tuberculosis. Halima Dawood has received speaker fees from SANOFI, unrelated to the study.  All other authors declare that they have no commercial or other associations that may pose a conflict of interest.

## FIGURE LEGENDS

Figure 1: Flow diagram of hospitalised SARS-CoV-2 infected patients screened and enrolled

Figure 2: Kaplan Meier plots of proportion of SARS-CoV-2 shedding by days following symptom onset by a) HIV status b) presence of obesity (BMI>30) c) CD4 T lymphocyte count amongst persons living with HIV (PLHIV) and d) HIV viral load amongst PLHIV

Figure 3: Kaplan Meier plots of proportion of high viral load SARS-CoV-2 shedding by days following symptom onset by a) HIV status b) HIV viral load in persons living with HIV

Figure 4: Spike protein antibody binding by HIV status by day 7,14 and 21 post symptom onset amongst hospitalised SARS-CoV-2 infected participants (n=201)

Table 1: Characteristics of persons hospitalised with SARS-CoV-2 infection with at least 2 study visits, by HIV status, South Africa (n=257)

Table 2: Accelerated Weibull Regression for duration of SARS-CoV-2 N gene detection by RT-PCR among hospitalised patients, South Africa

Table 3: Accelerated Weibull Regression for time taken from onset of symptoms to SARS-CoV-2 RT-PCR N gene $C_t$-value of $\geq$30 among a subset of hospitalised patients with laboratory confirmed SARS-CoV-2 infection and initial study SARS-CoV-2 RT-PCR N gene $C_t$-value <30, South Africa (n=94)

Table 4: SARS-CoV-2 serology results by HIV-status at day 7, 14 and 21 post symptom onset, among hospitalised persons with laboratory-confirmed SARS-CoV-2, South Africa (n=201)

LIST TABLES

Table 3: Accelerated Weibull Regression for time taken from onset of symptoms to SARS-CoV-2 RT-PCR N gene $C_t$-value of $\geq$30 among a subset of hospitalised patients with laboratory confirmed SARS-CoV-2 and initial study SARS-CoV-2 RT-PCR N gene $C_t$-value <30, South Africa (n=94)

Table 4: SARS-CoV-2 serology results by HIV-status at day 7, 14 and 21 post symptom onset, among hospitalised persons with laboratory confirmed SARS-CoV-2, South Africa (n=201)

| | All | | HIV-uninfected | | Persons living with HIV | | p-value |
|---|---|---|---|---|---|---|---|
| | n | % | n | % | n | % | |
| **Characteristics on admission** | 257 | | 155 | | 102 | | |
| **Age in years (median and IQR[a])** | 46 | 35-58 | 49 | 37-64 | 41 | 34-52 | <0.001 |
| **Age category (n=253)** | | | 152 | | 101 | | |
| 18-34 years | 61 | 24 | 33 | 22 | 28 | 28 | |
| 35-59 years | 135 | 53 | 72 | 47 | 63 | 62 | <0.001 |
| 60+ years | 57 | 23 | 47 | 31 | 10 | 10 | |
| **Sex (n=254)** | | | | | | | |
| Female | 155 | 61 | 77 | 51 | 78 | 76 | <0.001 |
| Male | 99 | 39 | 75 | 49 | 24 | 24 | |
| **Race (n=233)** | | | | | | | |
| Black African | 201 | 86 | 103 | 77 | 98 | 98 | |
| Coloured | 20 | 9 | 18 | 14 | 2 | 2 | <0.001 |
| Asian/Indian | 11 | 5 | 11 | 8 | 0 | 0 | |
| Other | 1 | 0 | 1 | 1 | 0 | 0 | |
| **Comorbidities** | | | | | | | |
| Chronic cardiac conditions (n=232) | 14 | 6 | 8 | 6 | 6 | 6 | 0.989 |
| Hypertension (n=232) | 77 | 33 | 53 | 40 | 24 | 24 | 0.013 |
| Diabetes (n=232) | 48 | 21 | 37 | 28 | 11 | 11 | 0.002 |
| Chronic kidney disease (n=230) | 5 | 2 | 4 | 3 | 1 | 1 | 0.293 |
| Obesity (n=232) | 38 | 16 | 25 | 19 | 13 | 13 | 0.249 |
| Current Tuberculosis (n=231) | 18 | 8 | 2 | 2 | 16 | 16 | <0.001 |
| **Smoking history (n=166)** | | | | | | | |

| | | | | | | | |
|---|---|---|---|---|---|---|---|
| Current smoker | 21 | 13 | 12 | 13 | 9 | 12 | |
| Former smoker | 17 | 10 | 8 | 9 | 9 | 12 | 0.805 |
| Non-smoker | 128 | 77 | 70 | 78 | 58 | 76 | |
| **Days from symptom onset to admission (median and IQR[a])** | 3 | 1-5 | 3 | 1-5 | 2 | 1-6 | 0.281 |
| **Disease severity on admission (WHO severity of COVID-19, qSOFA score) (n=214)** | | | 121 | | 93 | | |
| Mild | 39 | 18 | 20 | 17 | 19 | 20 | |
| Moderate | 137 | 64 | 76 | 63 | 61 | 66 | 0.401 |
| Severe | 38 | 18 | 25 | 21 | 13 | 14 | |
| **Signs and symptoms (n=243)** | | | 143 | | 100 | | |
| Fever | 111 | 45 | 68 | 48 | 43 | 43 | 0.483 |
| Cough | 192 | 79 | 113 | 79 | 79 | 79 | 0.849 |
| Dry | 115 | 60 | 73 | 65 | 42 | 53 | |
| Productive | 59 | 31 | 30 | 27 | 29 | 37 | 0.234 |
| Haemoptysis | 18 | 9 | 10 | 9 | 8 | 10 | |
| Sore throat | 79 | 33 | 50 | 35 | 29 | 29 | 0.329 |
| Chest Pain | 153 | 63 | 94 | 66 | 59 | 59 | 0.285 |
| Myalgia | 77 | 32 | 47 | 33 | 30 | 30 | 0.636 |
| Malaise | 127 | 52 | 76 | 53 | 51 | 51 | 0.742 |
| Dyspnoea | 160 | 66 | 95 | 66 | 65 | 65 | 0.817 |
| Confusion | 13 | 5 | 7 | 5 | 6 | 6 | 0.706 |
| Headache | 66 | 27 | 38 | 27 | 28 | 28 | 0.806 |
| Diarrhoea | 49 | 20 | 25 | 17 | 24 | 24 | 0.213 |
| **Characteristics during hospital admission** | | | | | | | |
| **Days in hospital (median and IQR[a])** | 9 | 6-13 | 8 | 5-13 | 9 | 6-15 | 0.118 |
| **Oxygen therapy required (n=235)** | 125 | 53 | 73 | 50 | 52 | 58 | 0.359 |

| | | | | | | | |
|---|---|---|---|---|---|---|---|
| **Invasive ventilation required (n=230)** | 11 | 5 | 6 | 4 | 5 | 6 | 0.571 |
| **Non-invasive ventilation required (n=227)** | 15 | 7 | 11 | 8 | 4 | 5 | 0.372 |
| **ICU[b] admission (n=235)** | 23 | 10 | 11 | 8 | 12 | 13 | 0.137 |
| **Glucocorticoids use (250)** | 84 | 34 | 51 | 34 | 33 | 34 | 0.616 |
| **In-hospital outcome (n=249)** | | | | | | | |
| Discharged | 211 | 85 | 126 | 83 | 85 | 87 | |
| Transferred | 19 | 8 | 14 | 9 | 5 | 5 | 0.475 |
| Died | 19 | 8 | 11 | 7 | 8 | 8 | |

Footnote: [a]IQR: interquartile range; [b]ICU: intensive care unit

| Characteristic | Number included in each category survival analysis | Duration of shedding (median) in days | Univariate analysis | | | Multivariable analysis | | |
|---|---|---|---|---|---|---|---|---|
| | | | Hazard ratio | 95% Confidence interval | p-value | Adjusted Hazard ratio | 95% Confidence interval | p-value |
| **Overall** | | 13 (6-25) | | | | | | |
| **Age category (years) (n=253)** | | | | | | | | |
| 18-34 years | 61 | 13 (2-32) | reference | | | | | |
| 35-59 years | 135 | 13 (7-24) | 0.86 | 0.60-1.23 | 0.417 | 1.01 | 0.65-1.56 | 0.973 |
| 60+ years | 57 | 13 (8-25) | 0.91 | 0.59-1.42 | 0.691 | 1.07 | 0.62-1.84 | 0.815 |
| **Sex (n=254)** | | | | | | | | |
| Male | 99 | 13 (6-24) | 1.08 | 0.79-1.48 | 0.622 | 1.01 | 0.69-1.49 | 0.945 |
| Female | 155 | 13 (6-29) | reference | | | | | |
| **HIV-status (n=257)** | | | | | | | | |
| HIV-uninfected | 155 | 15 (8-27) | reference | | | | | |
| HIV-infected | 102 | 11 (5-22) | 1.22 | 0.90-1.66 | 0.208 | | | |
| **HIV immunosuppression (n=257)** | | | | | | | | |
| HIV-uninfected | 155 | 15 (8-27) | referenc | | | referenc | | |

| | | | | | | | | |
|---|---|---|---|---|---|---|---|---|
| | | | e | | | e | | |
| HIV-infected CD4[a]≥200 | 41 | 11 (7-17) | 1.29 | 0.82-2.04 | 0.266 | 1.47 | 0.87-2.47 | 0.149 |
| HIV-infected CD4[a]<200 | 38 | 12 (4-29) | 1.06 | 0.70-1.61 | 0.792 | 0.98 | 0.61-1.58 | 0.935 |
| HIV-infected CD4[a] unknown | 23 | 10 (6-30) | 1.50 | 0.88-2.54 | 0.135 | 1.42 | 0.75-2.67 | 0.280 |
| **HIV-viral load category (n=257)** | | | | | | | | |
| HIV-uninfected | 155 | 15 (8-27) | reference | | | | | |
| HIV-infected viral load <400copies/ml | 37 | 13 (6-17) | 1.57 | 0.98-2.50 | 0.058 | | | |
| HIV-infected viral load ≥400 copies/ml | 27 | 13 (4-37) | 0.93 | 0.56-1.54 | 0.772 | | | |
| HIV-infected viral load unknown | 38 | 10 (5-25) | 1.26 | 0.82-1.92 | 0.293 | | | |
| **Glucocorticoid use (n=247)** | | | | | | | | |
| Yes | 84 | 16 (8-28) | 0.96 | 0.69-1.32 | 0.787 | | | |
| No | 163 | 13 (6-24) | reference | | | | | |
| **Severity of illness (n=214)** | | | | | | | | |
| Mild | 39 | 15 (6-31) | reference | | | reference | | |
| Moderate | 137 | 13 (7-24) | 1.20 | 0.78-1.85 | 0.403 | 1.24 | 0.80-1.92 | 0.343 |
| Severe | 38 | 15 (4-25) | 0.97 | 0.55-1.74 | 0.929 | 1.03 | 0.58-1.84 | 0.92 |

| | | | | | | | | |
|---|---|---|---|---|---|---|---|---|
| **Specific comorbidities** | | | | | | | | |
| Hypertension (n=232) | 77 | 13 (8-27) | 0.98 | 0.70-1.37 | 0.923 | | | |
| no hypertension | 155 | 15 (7-27) | reference | | | | | |
| Diabetes (n=232) | 48 | 15 (10-24) | 0.84 | 0.56-1.25 | 0.395 | | | |
| no diabetes | 184 | 13 (6-28) | reference | | | | | |
| Obesity (n=232) | 38 | 18 (11-34) | 0.58 | 0.38-0.90 | 0.016 | 0.55 | 0.33-0.90 | 0.018 |
| no obesity | 194 | 13 (6-25) | reference | | | | | |
| Tuberculosis (231) | 18 | 12 (4-27) | 1.30 | 0.75-2.26 | 0.341 | | | |
| no tuberculosis | 213 | 14 (7-27) | reference | | | | | |
| **Smoking history (n=166)** | | | | | | | | |
| Current smoker | 21 | 9 (1-20) | reference | | | | | |
| Former smoker | 17 | 15 (8-21) | 0.68 | 0.31-1.51 | 0.346 | | | |
| Non-smoker | 128 | 14 (7-28) | 0.63 | 0.36-1.11 | 0.112 | | | |
| **SARS-CoV-2 PCR positive on stool samples by day 7 post symptom onset (n=200)** | | | | | | | | |
| No | 132 | 11 (4-20) | reference | | | | | |
| Yes | 68 | 16 (9-29) | 0.65 | 0.45-0.93 | 0.021 | | | |
| **SARS-CoV-2 PCR positive on stool samples at day 14 post symptom onset (n=156)** | | | | | | | | |
| No | 113 | 13 (6-21) | referenc | | | | | |

| | | | | | |
|---|---|---|---|---|---|
| | | | e | | |
| Yes | 43 | 22 (13-37) | 0.40 | 0.26-0.63 | <0.001 |
| **Anti-spike protein antibodies by day 7 post symptom onset (n=201)** | | | | | |
| No | 40 | 10 (3-33) | referenc e | | |
| Yes | 161 | 13 (6-23) | 1.17 | 0.76-1.79 | 0.477 |
| **Anti-spike protein antibodies at day 14 post symptom onset (n=156)** | | | | | |
| No | 21 | 12 (3-37) | referenc e | | |
| Yes | 135 | 15 (8-25) | 1.32 | 0.75-2.33 | 0.330 |

Footnote: [a]CD4 – CD4 T lymphocyte count in cells/µl

Table 3: Accelerated Weibull Regression for time taken from onset of symptoms to SARS-CoV-2 RT-PCR N gene $C_t$-value of ≥30 among a subset of hospitalised patients with laboratory confirmed SARS-CoV2 and initial study SARS-CoV-2 RT-PCR N gene $C_t$-value <30, South Africa (n=94)

| **Characteristic** | **Number included in each category survival analysis** | **Median time from symptom onset to SARS-CoV-2 N gene $C_t$ value≥30** | **Univariate analysis** | | | **Multivariable analysis** | | |
|---|---|---|---|---|---|---|---|---|
| | | | **Hazard ratio** | **95% Confidence interval** | **p-value** | **Hazard ratio** | **95% Confidence interval** | **p-value** |
| **Overall** | | 8 (4-17) | | | | | | |
| **Age category (years) (n=93)** | | | | | | | | |
| 18-34 years | 22 | 7 (3-22) | reference | | | | | |
| 35-59 years | 48 | 8 (4-15) | 1.51 | 0.86-2.63 | 0.150 | 1.60 | 0.88-2.91 | 0.126 |
| 60+ years | 23 | 10 (6-16) | 1.51 | 0.8-2.83 | 0.201 | 0.61 | 0.3-1.25 | 0.175 |
| **Sex (n=94)** | | | | | | | | |
| Male | 42 | 6 (4-13) | 1.42 | 0.91-2.20 | 0.120 | | | |
| Female | 52 | 9 (5-21) | reference | | | | | |
| **Immunocompromised category** | | | | | | | | |
| HIV-uninfected | 53 | 7 (4-13) | reference | | | | | |
| HIV-infected CD4[a]≥200 | 14 | 7 (4-10) | 1.44 | 0.77-2.68 | 0.248 | 1.14 | 0.56-2.31 | 0.713 |

| | | | | | | | | |
|---|---|---|---|---|---|---|---|---|
| HIV-infected CD4[a]<200 | 18 | 27 (8-43) | 0.23 | 0.13-0.43 | <0.001 | 0.14 | 0.07-0.28 | <0.001 |
| HIV-infected CD4[a] unknown | 9 | 8 (1-19) | 0.59 | 0.26-1.30 | 0.188 | 0.71 | 0.3-1.7 | 0.441 |
| **HIV-viral load category** | | | | | | | | |
| HIV-uninfected | 53 | 7 (4-13) | reference | | | | | |
| HIV-infected viral load <400copies/ml | 11 | 6 (4-8) | 1.77 | 0.88-3.56 | 0.107 | | | |
| HIV-infected viral load >400 copies/ml | 13 | 26 (10-41) | 0.34 | 0.18-0.64 | 0.001 | | | |
| HIV-infected viral load unknown | 17 | 13 (4-26) | 0.30 | 0.15-0.57 | <0.001 | | | |
| **Glucocorticoid use (89)** | | | | | | | | |
| Yes | 32 | 12 (6-24) | 0.92 | 0.59-1.44 | 0.718 | 1.20 | 0.71-2.04 | 0.490 |
| No | 57 | 7 (4-13) | reference | | | | | |
| **Severity of illness (n=75)** | | | | | | | | |
| Mild | 13 | 13 (8-23) | reference | | | | | |
| Moderate | 48 | 7 (4-15) | 1.60 | 0.82-3.10 | 0.165 | | | |
| Severe | 14 | 9 (5-37) | 1.17 | 0.53-2.62 | 0.694 | | | |
| **Specific comorbidities (n=84)** | | | | | | | | |
| Hypertension | 25 | 8 (6-16) | 1.42 | 0.87-2.31 | 0.164 | | | |
| no hypertension | 59 | 9 (5-19) | reference | | | | | |
| Diabetes | 16 | 6 (3-13) | 0.96 | 0.52-1.77 | 0.888 | | | |
| no diabetes | 68 | 9 (5-19) | reference | | | | | |
| Tuberculosis | 9 | 20 (8-37) | 0.67 | 0.34-1.35 | 0.268 | | | |
| no tuberculosis | 75 | 8 (5-16) | reference | | | | | |
| Obesity | 16 | 7 (4-20) | 1.07 | 0.60-1.92 | 0.809 | | | |
| No obesity | 68 | 9 (5-17) | reference | | | | | |
| **Smoking history (n=62)** | | | | | | | | |
| Current smoker | 7 | 13 (1-29) | reference | | | | | |

| | | | | | |
|---|---|---|---|---|---|
| Former smoker | 7 | 11 (6-24) | 0.87 | 0.29-2.58 | 0.798 |
| Non-smoker | 48 | 8 (5-17) | 0.99 | 0.42-2.32 | 0.976 |
| **Spike ODB ≥0.4 by day 7 post symptom onset (n=76)** | | | | | |
| No | 21 | 10 (6-20) | reference | | |
| Yes | 55 | 6 (4-11) | 2.10 | 1.19-3.66 | 0.010 |
| **Spike ODB ≥0.4 at day 14 post symptom onset (n=56)** | | | | | |
| No | 9 | 37 (20-62) | reference | | |
| Yes | 47 | 7 (4-14) | 6.64 | 2.86-15.39 | <0.001 |

Footnote: [a]CD4 – CD4 T lymphocyte count in cells/µl

| | **Number with anti-spike protein antibodies 7 days post symptom onset** | | | **Number with anti-spike protein antibodies 14 days post symptom onset** | | | **Number with anti-spike protein antibodies 21 days post symptom onset** | | |
|---|---|---|---|---|---|---|---|---|---|
| | **n/N (%)** | **Odds ratio (95% CI)** | **p-value** | **n/N (%)** | **Odds ratio (95% CI)** | **p-value** | **n/N (%)** | **Odds ratio (95% CI)** | **p-value** |
| **All participants** | **161/201 (80)** | | | **135/156 (87)** | | | **106/120 (88)** | | |
| **HIV-uninfected** | 102/121 (84) | Reference | | 84/89 (94) | Reference | | 63/67 (94) | Reference | |
| **People living with HIV -CD4 count <200cells/µl** | 17/29 (59) | 0.26 (0.11-0.64) | 0.003 | 16/27 (59) | 0.09 (0.03-0.28) | <0.001 | 13/21 (62) | 0.10 (0.03-0.39) | 0.001 |
| **People** | 27/34 | 0.72 (0.27- | 0.50 | 20/23 | 0.40 (0.09- | 0.2 | 18/19 | 1.14 (0.12- | 0.9 |

| | | | | | | | | | |
|---|---|---|---|---|---|---|---|---|---|
| **living with HIV -CD4 count ≥200cells/µl** | (79) | 1.89) | 2 | (87) | 1.80) | 31 | (95) | 10.88) | 08 |
| **People living with HIV -CD4 count unknown** | 15/17 (88) | 1.4 (0.30-6.61) | 0.673 | 15/17 (88) | 0.45 (0.08-2.52) | 0.361 | 12/13 (92) | 0.76 (0.08-7.42) | 0.815 |

Footnote: CI confidence interval; CD4 count CD4 T-lymphocyte count

Figure 1

Accepted Manu

Figure 2

Accepted Manu

Figure 3

Accepted Manu

Figure 4

## Supplementary Material

ciac077_suppl_Supplementary_Appendix

ciac077_suppl_Supplementary_Figure_S1

ciac077_suppl_Supplementary_Legends

