# Prolonged Shedding of Severe acute respiratory syndrome coronavirus 2 (SARS-CoV-2) at High Viral Loads Among Hospitalized Immunocompromised Persons Living With Human Immunodeficiency Virus (HIV), South Africa

**DOI:** 10.1093/cid/ciac077

**Published:** 2022-02-02

**Authors:** Susan Meiring, Stefano Tempia, Jinal N Bhiman, Amelia Buys, Jackie Kleynhans, Mvuyo Makhasi, Meredith McMorrow, Jocelyn Moyes, Vanessa Quan, Sibongile Walaza, Mignon du Plessis, Nicole Wolter, Anne von Gottberg, Cheryl Cohen, COVID-19 shedding study group, John Black, Dominique Goedhals, Bonnie Maloba, Samantha Potgieter, Marianne Black, Vindana Chibabhai, Nonhlanhla Mbenenge, Trusha Nana, Sarah Stacey, Florette Treurnicht, Masego Moncho, Maphoshane Nchabeleng, Grace Shikwambane-Ntlemo, Rispah Chomba, Jeremy Nel, Anwar Hoosen, Mohamed Said, Junaid Bayat, Lisha Sookan, Surendra Sirkar, Halima Dawood, Sumayya Haffejee, Somasundram Pillay, Praksha Ramjathan, Nomonde Mvelase, Javid Mulla, Ruth Lekalakala-Mokaba, Matamela Madua, Sindile Ntuli, Thomas Crede, Kessendri Reddy, Jantjie Taljaard, Andrew Whitelaw

**Affiliations:** Division of Public Health Surveillance and Response, National Institute for Communicable Diseases, a Division of the National Health Laboratory Service, Johannesburg, South Africa; School of Public Health, Faculty of Health Sciences, University of the Witwatersrand, Johannesburg, South Africa; Centre for Respiratory Diseases and Meningitis, National Institute for Communicable Diseases, a Division of the National Health Laboratory Service, Johannesburg, South Africa; Influenza Division, Centers for Disease Control and Prevention, Atlanta, Georgia, USA; School of Public Health, Faculty of Health Sciences, University of the Witwatersrand, Johannesburg, South Africa; Centre for Respiratory Diseases and Meningitis, National Institute for Communicable Diseases, a Division of the National Health Laboratory Service, Johannesburg, South Africa; School of Pathology, Faculty of Health Sciences, University of the Witwatersrand, Johannesburg, South Africa; Centre for Respiratory Diseases and Meningitis, National Institute for Communicable Diseases, a Division of the National Health Laboratory Service, Johannesburg, South Africa; Centre for Respiratory Diseases and Meningitis, National Institute for Communicable Diseases, a Division of the National Health Laboratory Service, Johannesburg, South Africa; School of Public Health, Faculty of Health Sciences, University of the Witwatersrand, Johannesburg, South Africa; Centre for Respiratory Diseases and Meningitis, National Institute for Communicable Diseases, a Division of the National Health Laboratory Service, Johannesburg, South Africa; Influenza Division, Centers for Disease Control and Prevention, Atlanta, Georgia, USA; Division of Viral Diseases, US Centers for Disease Control and Prevention, Atlanta, Georgia, USA; Centre for Respiratory Diseases and Meningitis, National Institute for Communicable Diseases, a Division of the National Health Laboratory Service, Johannesburg, South Africa; Division of Public Health Surveillance and Response, National Institute for Communicable Diseases, a Division of the National Health Laboratory Service, Johannesburg, South Africa; Centre for Respiratory Diseases and Meningitis, National Institute for Communicable Diseases, a Division of the National Health Laboratory Service, Johannesburg, South Africa; School of Public Health, Faculty of Health Sciences, University of the Witwatersrand, Johannesburg, South Africa; Centre for Respiratory Diseases and Meningitis, National Institute for Communicable Diseases, a Division of the National Health Laboratory Service, Johannesburg, South Africa; School of Pathology, Faculty of Health Sciences, University of the Witwatersrand, Johannesburg, South Africa; Centre for Respiratory Diseases and Meningitis, National Institute for Communicable Diseases, a Division of the National Health Laboratory Service, Johannesburg, South Africa; School of Pathology, Faculty of Health Sciences, University of the Witwatersrand, Johannesburg, South Africa; Centre for Respiratory Diseases and Meningitis, National Institute for Communicable Diseases, a Division of the National Health Laboratory Service, Johannesburg, South Africa; School of Pathology, Faculty of Health Sciences, University of the Witwatersrand, Johannesburg, South Africa; Centre for Respiratory Diseases and Meningitis, National Institute for Communicable Diseases, a Division of the National Health Laboratory Service, Johannesburg, South Africa; School of Public Health, Faculty of Health Sciences, University of the Witwatersrand, Johannesburg, South Africa

**Keywords:** COVID-19, HIV, immunocompromised, respiratory virus, shedding duration

## Abstract

**Background:**

We assessed severe acute respiratory syndrome coronavirus 2 (SARS-CoV-2) RNA shedding duration and magnitude among persons living with human immunodeficiency virus (HIV, PLHIV).

**Methods:**

From May through December 2020, we conducted a prospective cohort study at 20 hospitals in South Africa. Adults hospitalized with symptomatic coronavirus disease 2019 (COVID-19) were enrolled and followed every 2 days with nasopharyngeal/oropharyngeal (NP/OP) swabs until documentation of cessation of SARS-CoV-2 shedding (2 consecutive negative NP/OP swabs). Real-time reverse transcription-polymerase chain reaction testing for SARS-CoV-2 was performed, and cycle-threshold (Ct) values < 30 were considered a proxy for high SARS-CoV-2 viral load. Factors associated with prolonged shedding were assessed using accelerated time-failure Weibull regression models.

**Results:**

Of 2175 COVID-19 patients screened, 300 were enrolled, and 257 individuals (155 HIV-uninfected and 102 PLHIV) had > 1 swabbing visit (median 5 visits [range 2–21]). Median time to cessation of shedding was 13 days (interquartile range [IQR] 6–25) and did not differ significantly by HIV infection. Among a subset of 94 patients (41 PLHIV and 53 HIV-uninfected) with initial respiratory sample C_t_-value < 30, median time of shedding at high SARS-CoV-2 viral load was 8 days (IQR 4–17). This was significantly longer in PLHIV with CD4 count < 200 cells/µL, compared to HIV-uninfected persons (median 27 days [IQR 8–43] vs 7 days [IQR 4–13]; adjusted hazard ratio [aHR] 0.14, 95% confidence interval [CI] .07–.28, *P* < .001), as well as in unsuppressed-HIV versus HIV-uninfected persons.

**Conclusions:**

Although SARS-CoV-2 shedding duration did not differ significantly by HIV infection, among a subset with high initial SARS-CoV-2 viral loads, immunocompromised PLHIV shed SARS-CoV-2 at high viral loads for longer than HIV-uninfected persons. Better HIV control may potentially decrease transmission time of SARS-CoV-2.

## Teaser

Severe acute respiratory syndrome coronavirus 2 (SARS-CoV-2) shedding duration did not significantly differ by human immunodeficiency virus (HIV) infection. Among a subset of persons with high initial SARS-CoV-2 viral load, immunocompromised persons living with HIV shed SARS-CoV-2 at higher viral loads for a longer duration, potentially increasing their transmission period.

Immunocompromised persons are thought to shed severe acute respiratory syndrome coronavirus 2 (SARS-CoV-2) for a longer duration, increasing time for viral transmission and potentially driving within host viral evolution [1–3]. South Africa has 7 500 000 persons living with human immunodeficiency virus (HIV, PLHIV), and there are no systematically collected data on duration of SARS-CoV-2 shedding in PLHIV. We hypothesized that PLHIV may shed SARS-CoV-2 for a longer period of time and at a higher viral load than HIV-uninfected individuals.

SARS-CoV-2 shedding from the upper respiratory tract extends for a mean of 17 days (15.5–18.6) [4]. Unlike severe acute respiratory syndrome coronavirus 1 (SARS-CoV-1), viral shedding of SARS-CoV-2 from the upper respiratory tract peaks on or before symptom onset, allowing some viral transmission to occur before symptom onset in infected individuals [4–8]. Quantifying SARS-CoV-2 viral load (Log_10_ RNA copies/mL) is calculated by converting qualitative real-time reverse transcription-polymerase chain reaction (rRT-PCR) cycle threshold values using calibration curves based on quantified E-gene in vitro RNA transcripts [9]. However, studies on SARS-CoV-2 infectiousness indicate that successful virus isolation is most likely from specimens with a rRT-PCR cycle threshold (Ct) value < 34(~7 Log_10_ copies/mL); however, not all of these specimens may be positive for viable virus [9, 10]. Using this threshold, infectiousness declines significantly 8 days after becoming symptomatic, even though SARS-CoV-2 viral RNA persistence has been shown to occur for months in some individuals [9, 11, 12].

Various factors have been associated with increased shedding duration, including increased age, male sex, severity of illness and use of corticosteroids [13, 14]. There are several published case reports indicating prolonged SARS-CoV-2 transmission in immunocompromised persons, mostly with cancers or autoimmune conditions, and more recently in an immunocompromised person living with HIV [15–18].

We aimed to evaluate the overall duration of SARS-CoV-2 shedding in upper respiratory tract specimens and duration of high viral load shedding in a cohort of PLHIV and HIV-uninfected persons hospitalized with COVID-19 in South Africa. We also estimated SARS-CoV-2 shedding duration in stool and blood specimens, and described serologic responses to SARS-CoV-2 infection.

## METHODS

### Study Design and Setting

We conducted a prospective cohort study from 1 May through 31 December 2020 (spanning the first wave and beginning of the second wave of the COVID-19 pandemic in South Africa). Persons hospitalized for symptomatic COVID-19 at one of 20 hospitals situated in 8 of the 9 South African provinces were invited to participate in the study if they met the following inclusion criteria: aged 18 years and older, laboratory confirmed diagnosis of SARS-CoV-2 in the previous 5 days, resided within a 50-kilometer radius of the hospital, and had laboratory confirmation of their HIV status.

Using standardized case report forms, demographic and clinical details were collected at enrollment, daily while in hospital, and at discharge from hospital/cessation of shedding/death of the participant. Included variables are detailed in Supplementary Material.

At enrollment and every second day thereafter, until cessation of shedding or death, a combined nasopharyngeal/oropharyngeal (NP/OP) swab was collected by trained nursing staff, using 2 flocked nylon plastic shaft swabs (1 for the nasopharynx and 1 for the oropharynx), which were then inserted together into Universal Transport Medium and transported on ice to the laboratory. Sensitivity of the combined NP/OP swab has been shown to be 97% for detection of SARS-CoV-2 in the upper airways [19, 20]. A rectal swab or stool was collected from patients at the same time intervals and transported to the laboratory in sealed containers. Results are available in Supplementary Material. Patients were followed up at home if they were still SARS-CoV-2 positive on NP/OP swab on discharge from hospital.

Whole blood and serum specimens were taken on enrollment and at days 7, 14, and 21 post symptom-onset for SARS-CoV-2 testing and serology. Results for SARS-CoV-2 testing of blood samples are available in Supplementary Materials. All specimens were transported to the National Institute for Communicable Diseases (NICD) in Johannesburg for processing and testing.

### Laboratory Diagnostics

rRT-PCR for the qualitative detection of nucleic acid from SARS-CoV-2 was performed on NP/OP, stool/rectal swabs and blood specimens using the Allplex™ nCoV 2019 kit (Seegene, Seoul, South Korea). Specimens were considered positive for SARS-CoV-2 nucleic acids if the Ct was < 40 for ≥ 1 of 3 gene targets. A nucleocapsid gene (N gene) Ct value < 30 on NP/OP specimens was used as a proxy for a high viral load based on published data showing a high correlation between low Ct values (using various gene targets), high viral load and increased odds of shedding cultivable virus [10, 12, 21, 22].

Antibody detection against trimeric ectodomain HexaPro spike protein was performed on serum specimens as described previously [23, 24]. Absorbance at 450 nm was measured, and specimens with optical density (OD) > 0.4 were considered positive for anti-spike protein antibodies.

SARS-CoV-2 sequencing was performed on the first NP/OP specimens on enrollment from 10 randomly selected participants of 29, who demonstrated high viral load shedding for > 14 days. Clade and lineage assignments were made using the online Nextclade (https://clades.nextstrain.org/) and Pangolin (https://pangolin.cog-uk.io/) applications, which also enable identification of known variants of concern as well as novel mutations. See Supplementary Methods for more details.

### Definitions

Shedding was defined as presence of SARS-CoV-2 nucleic acid in a specimen as detected by a positive SARS-CoV-2 rRT-PCR result. Participants were deemed to have stopped shedding SARS-CoV-2 from the respiratory tract once 2 consecutive NP/OP swabs, taken at least 2 days apart, tested negative for all 3 gene targets on rRT-PCR. Time to cessation of shedding was taken from date of symptom onset to the date of the last rRT-PCR SARS-CoV-2 positive NP/OP swab prior to the two consecutive negative swabs. Participants were deemed to be shedding SARS-CoV-2 virus at high viral loads if their NP/OP swab N gene Ct value was < 30. Persistence of high viral load was measured in the subgroup of individuals whose first study NP/OP swab had an N gene Ct value < 30 and was measured from date of symptom onset to the last date where NP/OP swab N gene Ct value was < 30. PLHIV were deemed to be significantly immunocompromised if their CD4 T-lymphocyte count was < 200 cells/µL and not HIV virally suppressed if their HIV viral load measured > 400 copies/mL in the 3 months before hospital admission [25, 26]. Severity of COVID-19 was categorized using respiratory rate at time of admission according to the WHO clinical classification of COVID-19 and a quick Sequential (Sepsis-related) Organ Failure Assessment (qSOFA) score [27, 28].

### Statistical Analysis

Data were captured on a real-time data capture (REDCAP) database and transferred onto a password protected Microsoft Access database management system [29]. Statistical analysis was performed using Stata version 14 (StataCorp Inc., College Station, Texas, USA).

We used Kaplan-Meier estimates and Weibull accelerated failure time regression models to measure time from symptom onset to (i) cessation of shedding and (ii) low viral load shedding (at Ct values *>* 30). The Weibull model was chosen as it allows explanatory variables to proportionately increase or decrease the time to the end-point [30]. Hazard ratios measured the hazard of reaching the end-points with a ratio of < 1 indicating a longer duration of shedding or a longer duration of high viral load shedding. *P*-values < .2 on univariate analysis were included in all multivariable models, and nonsignificant variables (*P*-value > .05) were dropped using step-wise manual backward elimination. Patients with missing data were dropped from the models.

### Ethics

Ethics clearance for the study was obtained through various university health research ethics committees (HREC), namely, University of the Witwatersrand HREC (Medical) (M160667); Stellenbosch University HREC (15206); University of Pretoria HREC (256/2020), and University of the Free State HREC (HSD2020/0625). Permission to conduct the study was obtained from each Provincial Department of Health Research Committee. All participants gave written informed consent to participate in the study.

## RESULTS

### Participant Inclusion and Characterization

**Figure 1.**
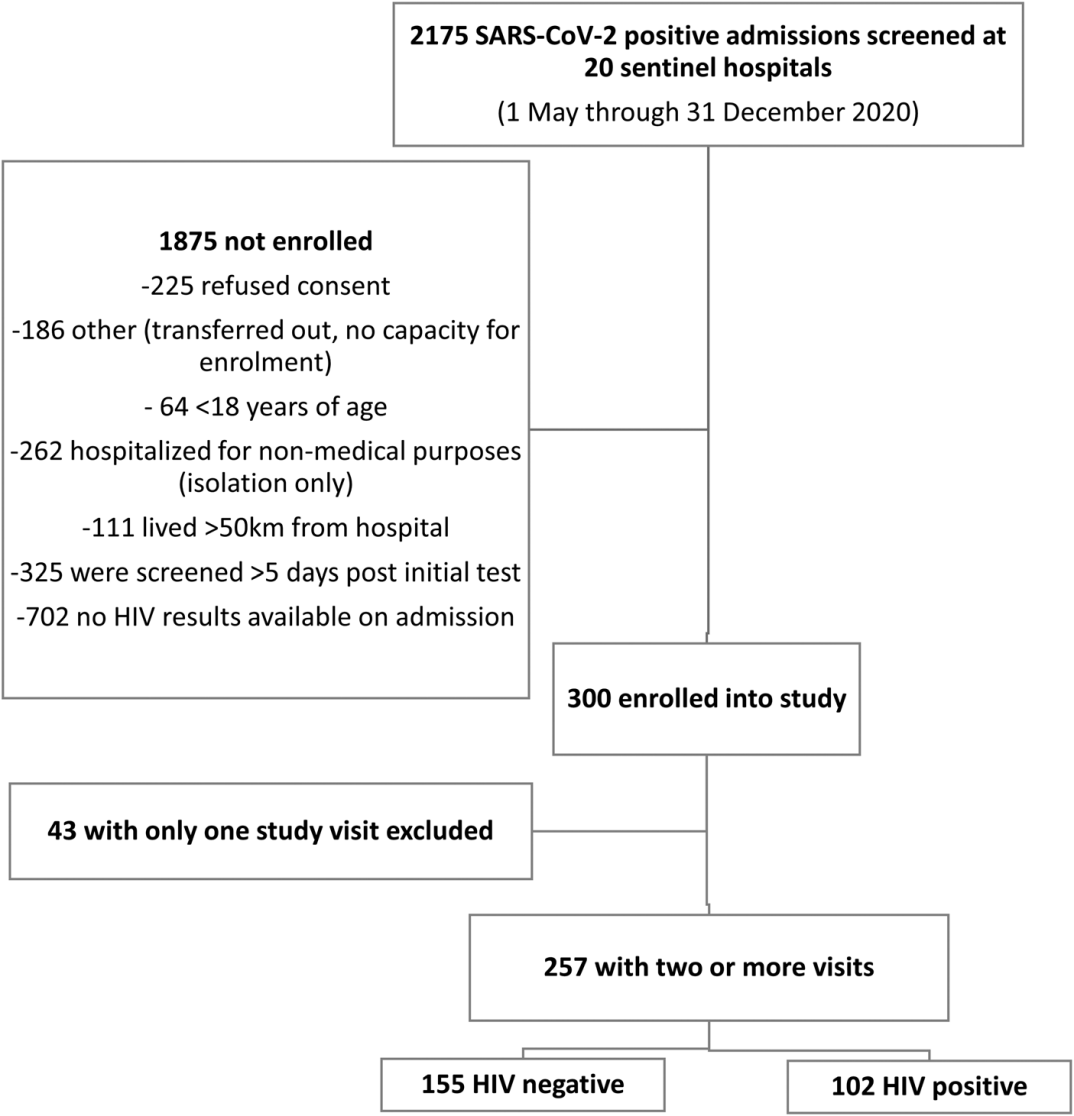
Flow diagram of hospitalized SARS-CoV-2-infected patients screened and enrolled. Abbreviations: HIV, human immunodeficiency virus; SARS-CoV-2, severe acute respiratory syndrome coronavirus 2.

From 1 May through 31 December 2020, 2175 hospitalized COVID-19 patients were screened for enrolment at 20 sentinel hospitals across South Africa (Figure 1). Of the 1875 not enrolled: 225 refused consent, 186 were transferred out of the hospital on the day of screening, 64 were minors, and 1400 did not meet the eligibility criteria (Figure 1 and Supplementary Table 1). Three hundred persons were enrolled into the study of which 257 had at least 2 study visits (range 2–21), with a median of 5 visits per participant (Supplementary Figure 1). Of these, 155 persons were HIV-uninfected and 102 PLHIV (Figure 1).

Of the 257 patients included in the cohort: median age was 46 years (IQR 35–58 years), PLHIV were significantly younger than HIV-uninfected persons (median of 41 years in PLHIV and 49 years in HIV-uninfected, *P* < .001). Sixty-one percent of participants were female with significantly more females than males represented among PLHIV (*P* < .001). The majority of participants (86%) were of Black African descent. Overall, 33% had underlying hypertension (40% HIV-uninfected vs 24% PLHIV, *P* = .01), 21% had diabetes (28% HIV-uninfected vs 11% PLHIV, *P* = .002), 16% were obese (19% HIV-uninfected vs 13% PLHIV, *P* = .25), and 8% had underlying tuberculosis (2% HIV-uninfected vs 16% PLHIV, *P* < .001). Thirteen percent were current cigarette smokers (Table 1). Of PLHIV, 84% were currently receiving antiretroviral therapy, median CD4 T-cell count was 221 cells/µL (IQR 48–443) (with 53% having CD4 T-cell count* > *200 cells/µL), and 59% were HIV virally suppressed (HIV viral load < 400 copies/mL).

**Table 1.** Characteristics of Persons Hospitalized With SARS-CoV-2 Infection With at Least 2 Study Visits, by HIV Status, South Africa (n = 257)

	All		HIV-uninfected		Persons Living With HIV		*P* value
	n	%	n	%	n	%	
Characteristics on admission	257		155		102		
Age in years (median and IQR)	46	35–58	49	37–64	41	34–52	<.001
Age category (n = 253)			152		101		
18–34 years	61	24	33	22	28	28	<.001
35–59 years	135	53	72	47	63	62	
≥60 years	57	23	47	31	10	10	
Sex (n = 254)							
Female	155	61	77	51	78	76	<.001
Male	99	39	75	49	24	24	
Race (n = 233)							
Black African	201	86	103	77	98	98	<.001
Mixed race	20	9	18	14	2	2	
Asian/Indian	11	5	11	8	0	0	
Other	1	0	1	1	0	0	
Comorbidities							
Chronic cardiac conditions (n = 232)	14	6	8	6	6	6	.989
Hypertension (n = 232)	77	33	53	40	24	24	.013
Diabetes (n = 232)	48	21	37	28	11	11	.002
Chronic kidney disease (n = 230)	5	2	4	3	1	1	.293
Obesity (n = 232)	38	16	25	19	13	13	.249
Current tuberculosis (n = 231)	18	8	2	2	16	16	<.001
Smoking history (n = 166)							
Current smoker	21	13	12	13	9	12	.805
Former smoker	17	10	8	9	9	12	
Nonsmoker	128	77	70	78	58	76	
Days from symptom onset to admission (median and IQR)	3	1–5	3	1–5	2	1–6	.281
Disease severity on admission (WHO severity of COVID-19, qSOFA score) (n = 214)			121		93		
Mild	39	18	20	17	19	20	.401
Moderate	137	64	76	63	61	66	
Severe	38	18	25	21	13	14	
Signs and symptoms (n = 243)			143		100		
Fever	111	45	68	48	43	43	.483
Cough	192	79	113	79	79	79	.849
Dry	115	60	73	65	42	53	.234
Productive	59	31	30	27	29	37	
Hemoptysis	18	9	10	9	8	10	
Sore throat	79	33	50	35	29	29	.329
Chest pain	153	63	94	66	59	59	.285
Myalgia	77	32	47	33	30	30	.636
Malaise	127	52	76	53	51	51	.742
Dyspnoea	160	66	95	66	65	65	.817
Confusion	13	5	7	5	6	6	.706
Headache	66	27	38	27	28	28	.806
Diarrhea	49	20	25	17	24	24	.213
Characteristics during hospital admission							
Days in hospital (median and IQR)	9	6–13	8	5–13	9	6–15	.118
Oxygen therapy required (n = 235)	125	53	73	50	52	58	.359
Invasive ventilation required (n = 230)	11	5	6	4	5	6	.571
Noninvasive ventilation required (n = 227)	15	7	11	8	4	5	.372
ICU admission (n = 235)	23	10	11	8	12	13	.137
Glucocorticoids use (250)	84	34	51	34	33	34	.616
In-hospital outcome (n = 249)							
Discharged	211	85	126	83	85	87	.475
Transferred	19	8	14	9	5	5	
Died	19	8	11	7	8	8	

Abbreviations: COVID-19, coronavirus disease 2019; HIV, human immunodeficiency virus; ICU, intensive care unit; IQR, interquartile range; qSOFA, quick Sequential (Sepsis-related) Organ Failure Assessment; SARS-CoV-2; severe acute respiratory syndrome coronavirus 2; WHO, World Health Organization.

Patients were admitted for a median of 9 days (IQR 6–13) in hospital. Eight participants had unknown outcome. Overall, 10% (24/250) of participants died, 8% (19) during their hospital admission, and a further 2% (5) within 2 months post-discharge. This did not differ significantly by HIV status. Of 214 participants with known parameters to assess disease severity, most (64%, 137/214) had moderate disease severity, with 18% (39/214) having mild and 18% (38/214) having severe COVID-19. HIV-uninfected persons and PLHIV had similar clinical presentation. The most common presenting symptoms were cough (79%, 192/243), dyspnea (66%, 160/243), chest pain (63%, 153/243), malaise (52%, 127/243), and fever (45%, 111/243). Oxygen therapy was required for 53% (125/235), noninvasive ventilation for 7% (15/227), and invasive ventilation for 5% (11/230). Ten percent (23/235) were admitted to an intensive care unit. Glucocorticoids were given to 34% (84/250) of individuals while in hospital (Table 1).

### Factors Associated With Duration of Shedding

**Figure 2.**
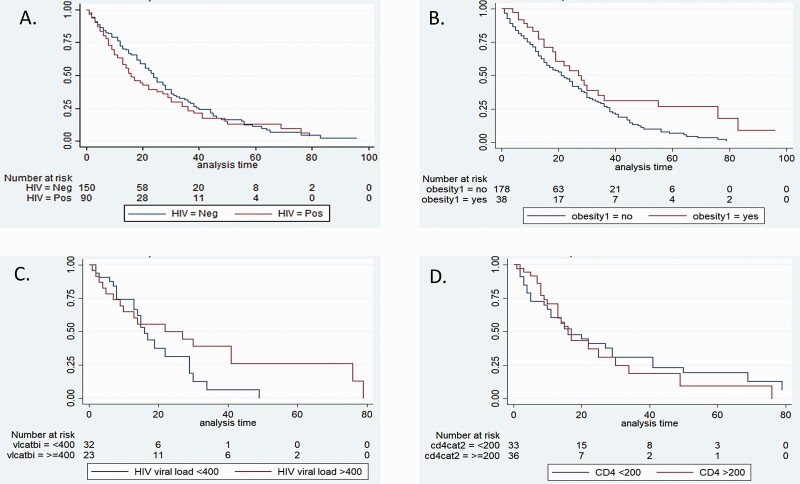
Kaplan-Meier plots of proportion of SARS-CoV-2 shedding by days following symptom onset by (*A*) HIV status, (*B*) presence of obesity (BMI > 30), (*C*) HIV viral load among PLHIV, and (*D*). CD4 T-lymphocyte count among persons living with HIV (PLHIV). Abbreviations: BMI, body mass index; HIV, human immunodeficiency virus; SARS-CoV-2, severe acute respiratory syndrome coronavirus 2.

Of the 257 participants with at least 2 swabbing visits: 186 were followed up until cessation of SARS-CoV-2 shedding, 14 died prior to stopping shedding, and 57 were lost to follow-up. Overall, there were 4881 patient days of observation. The median duration of SARS-CoV-2 shedding from the 257 participants was 13 days (IQR 6-–25) from symptom onset: 15 days (IQR 8–27) in HIV-uninfected versus 11 days (IQR 5–22) in PLHIV (adjusted hazard ratio [aHR)] 1.16, 95% confidence interval [CI] .8–1.68, *P* = .44). Duration of SARS-CoV-2 shedding was not significantly different by different age categories, sex, HIV immunosuppression, severity of illness, and use of glucocorticoids. However, after adjusting for confounders on multivariable analysis, the duration of SARS-CoV-2 detection was 45% longer in obese compared to non-obese persons (18 days [IQR 11–34] vs 13 days (IQR 6–25), aHR 0.55, 95%CI .33–.90, *P* = .02) (Table 2; Figure 2; Supplementary Table 2).

**Table 2.** Accelerated Weibull Regression for Duration of SARS-CoV-2 N Gene Detection by RT-PCR Among Hospitalized Patients, South Africa

Characteristic	Number Included in Each Category Survival Analysis	Duration of Shedding in days, Median (IQR)	Univariate Analysis			Multivariable Analysis		
			HR	95% CI	*P* value	Adjusted HR	95% CI	*P* value
Overall		13 (6–25)						
Age category (years) (n = 253)								
18–34 years	61	13 (2–32)	Reference					
35–59 years	135	13 (7–24)	0.86	.60–1.23	.417	1.01	.65–1.56	.973
≥60 years	57	13 (8–25)	0.91	.59–1.42	.691	1.07	.62–1.84	.815
Sex (n = 254)								
Male	99	13 (6–24)	1.08	.79–1.48	.622	1.01	.69–1.49	.945
Female	155	13 (6–29)	Reference					
HIV status (n = 257)								
HIV-uninfected	155	15 (8–27)	Reference					
PLHIV	102	11 (5–22)	1.22	.90–1.66	.208			
HIV immunosuppression (n = 257)								
HIV-uninfected	155	15 (8–27)	Reference			Reference		
PLHIV CD4^a^*≥*200	41	11 (7–17)	1.29	.82–2.04	.266	1.47	.87–2.47	.149
PLHIV CD4^a^ < 200	38	12 (4–29)	1.06	.70–1.61	.792	0.98	.61–1.58	.935
HIV-infected CD4^a^ unknown	23	10 (6–30)	1.50	.88–2.54	.135	1.42	.75–2.67	.280
HIV viral load category (n = 257)								
HIV-uninfected	155	15 (8–27)	Reference					
PLHIV viral load < 400 copies/mL	37	13 (6–17)	1.57	.98–2.50	.058			
PLHIV viral load ≥ 400 copies/mL	27	13 (4–37)	0.93	.56–1.54	.772			
HIV-infected viral load unknown	38	10 (5–25)	1.26	.82–1.92	.293			
Glucocorticoid use (n = 247)								
Yes	84	16 (8–28)	0.96	.69–1.32	.787			
No	163	13 (6–24)	Reference					
Severity of illness (n = 214)								
Mild	39	15 (6–31)	Reference			Reference		
Moderate	137	13 (7–24)	1.20	.78–1.85	.403	1.24	.80–1.92	.343
Severe	38	15 (4–25)	0.97	.55–1.74	.929	1.03	.58–1.84	.92
Specific comorbidities								
Hypertension (n = 232)	77	13 (8–27)	0.98	.70–1.37	.923			
no hypertension	155	15 (7–27)	Reference					
Diabetes (n = 232)	48	15 (10–24)	0.84	.56–1.25	.395			
No diabetes	184	13 (6–28)	Reference					
Obesity (n = 232)	38	18 (11–34)	0.58	.38–0.90	.016	0.55	.33.90	.018
No obesity	194	13 (6–25)	Reference					
Tuberculosis (231)	18	12 (4–27)	1.30	0.75–2.26	.341			
No tuberculosis	213	14 (7–27)	Reference					
Smoking history (n = 166)								
Current smoker	21	9 (1–20)	Reference					
Former smoker	17	15 (8–21)	0.68	.31–1.51	.346			
Nonsmoker	128	14 (7–28)	0.63	.36–1.11	.112			
SARS-CoV-2 PCR positive on stool samples by day 7 post symptom onset (n = 200)								
No	132	11 (4–20)	Reference					
Yes	68	16 (9–29)	0.65	.45–.93	.021			
SARS-CoV-2 PCR positive on stool samples at day 14 post symptom onset (n = 156)								
No	113	13 (6–21)	Reference					
Yes	43	22 (13–37)	0.40	.26–.63	<.001			
Anti-spike protein antibodies by day 7 post symptom onset (n = 201)								
No	40	10 (3–33)	Reference					
Yes	161	13 (6–23)	1.17	.76–1.79	.477			
Anti-spike protein antibodies at day 14 post symptom onset (n = 156)								
No	21	12 (3–37)	Reference					
Yes	135	15 (8–25)	1.32	.75–2.33	.330			

Abbreviations: CI, confidence interval; HIV, human immunodeficiency virus; HR, hazard ratio; IQR, interquartile range; RT-PCR, reverse transcription polymerase chain reaction; SARS-CoV-2; severe acute respiratory syndrome coronavirus 2.

CD4 – CD4 T-lymphocyte count in cells/µL.

### Factors Associated With Shedding Potentially Infectious Virus

**Figure 3.**
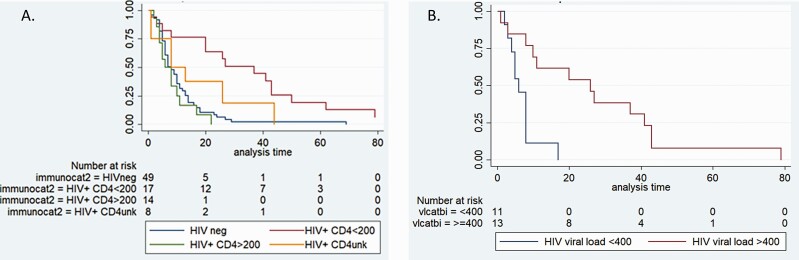
Kaplan-Meier plots of proportion of high viral load SARS-CoV-2 shedding by days following symptom onset by (*A*) HIV status and (*B*) HIV viral load in persons living with HIV. Abbreviations: HIV, human immunodeficiency virus; SARS-Cov-2, severe acute respiratory syndrome coronavirus 2.

Of a subset of 94 participants with N gene Ct value < 30 at enrolment (41 PLHIV and 53 HIV-uninfected persons), the median duration of SARS-CoV-2 shedding at N gene Ct value < 30 from symptom onset was 8 days (IQR 4–17). On multivariable analysis, when adjusting for age and glucocorticoid use, PLHIV with a CD4 cell count < 200 cells/µL shed at high SARS-CoV-2 viral loads for longer (median 27days, IQR 8–43, aHR 0.14, 95% CI .07–.28, *P* < .001), whereas PLHIV with CD4 count* > *200 cells/μL shed at high SARS-CoV-2 viral loads for a similar time period (median 7days, IQR 4–10, aHR 1.14, 95% CI .56–2.31, *P* = .71), compared to HIV-uninfected persons (median 7 days, IQR 4–13). Similarly, PLHIV with HIV viral loads* > *400 copies/mL were more likely to shed SARS-CoV-2 at high viral loads for longer (26 days, IQR 10–41, unadjusted HR 0.34, 95% CI .18–.64, *P* < .001) than PLHIV with HIV viral suppression (6 days, IQR 4–8, unadjusted HR 1.77, 95% CI .88–3.56, *P* = .107) and those who were HIV-uninfected (7 days, IQR 4–13) (Table 3; Figure 3; Supplementary Table 3).

**Table 3.** Accelerated Weibull Regression for Time Taken From Onset of Symptoms to SARS-CoV-2 RT-PCR N Gene Ct Value of ≥30 Among a Subset of Hospitalized Patients With Laboratory Confirmed SARS-CoV-2 and Initial Study SARS-CoV-2 RT-PCR N Gene Ct value < 30, South Africa (n = 94)

Characteristic	Number Included in Each Category Survival Analysis	Median Time From Symptom Onset to SARS-CoV-2 N gene Ct value* ≥*30 (IQR)	Univariate Analysis			Multivariable Analysis		
			HR	95% CI	*P* value	HR	95% CI	*P* value
Overall		8 (4–17)						
Age category (years) (n = 93)								
18–34 years	22	7 (3–22)	Reference					
35–59 years	48	8 (4–15)	1.51	0.86–2.63	.150	1.60	.88–2.91	.126
≥60years	23	10 (6–16)	1.51	0.8–2.83	.201	0.61	.3–1.25	.175
Sex (n = 94)								
Male	42	6 (4–13)	1.42	0.91–2.20	.120			
Female	52	9 (5–21)	Reference					
Immunocompromised category								
HIV-uninfected	53	7 (4–13)	Reference					
HIV-infected CD4^a^* ≥*200	14	7 (4–10)	1.44	.77–2.68	.248	1.14	.56–2.31	.713
HIV-infected CD4^a^ < 200	18	27 (8–43)	0.23	.13–.43	<.001	0.14	.07–.28	<.001
HIV-infected CD4^a^ unknown	9	8 (1–19)	0.59	.26–1.30	.188	0.71	.3–1.7	.441
HIV viral load category								
HIV-uninfected	53	7 (4–13)	Reference					
HIV-infected viral load < 400 copies/mL	11	6 (4–8)	1.77	.88–3.56	.107			
HIV-infected viral load ≥400 copies/mL	13	26 (10–41)	0.34	.18–.64	.001			
HIV-infected viral load unknown	17	13 (4–26)	0.30	.15–.57	<.001			
Glucocorticoid use (89)								
Yes	32	12 (6–24)	0.92	.59–1.44	.718	1.20	.71–2.04	.490
No	57	7 (4–13)	Reference					
Severity of illness (n = 75)								
Mild	13	13 (8–23)	Reference					
Moderate	48	7 (4–15)	1.60	.82–3.10	.165			
Severe	14	9 (5–37)	1.17	.53–2.62	.694			
Specific comorbidities (n = 84)								
Hypertension	25	8 (6–16)	1.42	.87–2.31	.164			
No hypertension	59	9 (5–19)	Reference					
Diabetes	16	6 (3–13)	0.96	.52–1.77	.888			
No diabetes	68	9 (5–19)	Reference					
Tuberculosis	9	20 (8–37)	0.67	.34–1.35	.268			
No tuberculosis	75	8 (5–16)	Reference					
Obesity	16	7 (4–20)	1.07	.60–1.92	.809			
No obesity	68	9 (5–17)	Reference					
Smoking history (n = 62)								
Current smoker	7	13 (1–29)	Reference					
Former smoker	7	11 (6–24)	0.87	.29–2.58	.798			
Nonsmoker	48	8 (5–17)	0.99	.42–2.32	.976			
Spike ODB > 0.4 by day 7 post symptom onset (n = 76)								
No	21	10 (6–20)	Reference					
Yes	55	6 (4–11)	2.10	1.19–3.66	.010			
Spike ODB > 0.4 at day 14 post symptom onset (n = 56)								
No	9	37 (20–62)	Reference					
Yes	47	7 (4–14)	6.64	2.86–15.39	<.001			

Abbreviations: CI, confidence interval; HIV, human immunodeficiency virus; HR, hazard ratio; RT-PCR, reverse transcription polymerase chain reaction; IQR, interquartile range; SARS-CoV-2; severe acute respiratory syndrome coronavirus 2.

CD4 – CD4 T-lymphocyte count in cells/µL.

### SARS-CoV-2 Antibodies

**Figure 4.**
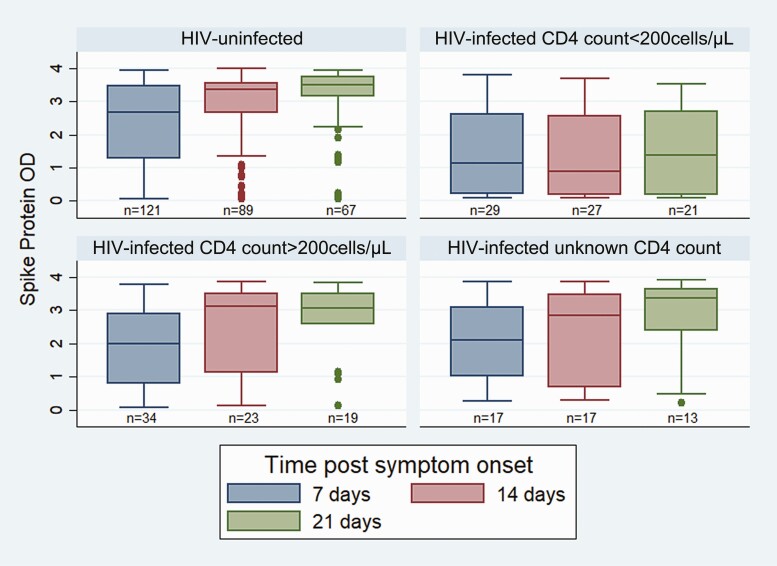
Spike protein antibody binding by HIV status by day 7, 14 and 21, post symptom onset amongst hospitalized SARS-CoV-2-infected participants (n = 201). Abbreviations: HIV, human immunodeficiency virus; SARS-CoV-2, severe acute respiratory syndrome coronavirus 2.

The proportion of participants with positive anti-spike protein antibodies increased from 80% (161/201) by day 7 to 87% (135/156) at day 14 post-symptom onset. PLHIV with CD4 count < 200 cells/μL were significantly less likely to develop anti-spike protein antibody titers by day 7, 14, or 21 post symptom-onset than HIV-uninfected persons (Table 4). Median anti-spike protein antibody titers were significantly higher by 7 and 14 days post-symptom onset in HIV-uninfected persons (Figure 4). By day 21, there were 4 HIV-uninfected and 10 PLHIV (8 with CD4 counts < 200 cells/µL, 1 with CD4 count > 200 cells/µL and 1 with unknown CD4 count) who did not develop anti-spike protein antibodies. Seven (2 HIV-uninfected and 5 PLHIV with CD4 count < 200 cells/µL) of these 14 shed SARS-CoV-2 viral RNA from the upper respiratory tract for 20 days or longer post symptom-onset (all 5 PLHIV still had N gene Ct values < 26 by day 20).

**Table 4.** SARS-CoV-2 Serology Results by HIV-status at Day 7, 14, and 21 Post Symptom Onset, Among Hospitalized Persons with Laboratory Confirmed SARS-CoV-2, South Africa (n = 201)

	Number with anti-spike protein antibodies			Number with anti-spike protein antibodies			Number with anti-spike protein antibodies		
	7 days			14 days			21 days		
	post symptom onset			post symptom onset			post symptom onset		
	n/N (%)	Odds ratio (95% CI)	*P* value	n/N (%)	Odds ratio (95% CI)	*P* value	n/N (%)	Odds ratio (95% CI)	*P* value
**All participants**	**161/201 (80)**			**135/156 (87)**			**106/120 (88)**		
**HIV-uninfected**	102/121 (84)	Reference		84/89 (94)	Reference		63/67 (94)	Reference	
**Persons living with HIV CD4** ^**a**^ **count < 200 cells/µL**	17/29 (59)	0.26 (.11–.64)	.003	16/27 (59)	0.09 (.03–.28)	<.001	13/21 (62)	0.10 (.03–.39)	.001
**Persons living with HIV CD4** ^**a**^ **count ≥200cells/µL**	27/34 (79)	0.72 (.27–1.89)	.502	20/23 (87)	0.40 (.09–1.80)	.231	18/19 (95)	1.14 (.12–10.88)	.908
**Persons living with HIV CD4** ^**a**^ **count unknown**	15/17 (88)	1.4 (.30–6.61)	.673	15/17 (88)	0.45 (.08–2.52)	.361	12/13 (92)	0.76 (.08–7.42)	.815

Abbreviations: CI confidence interval; HIV, human immunodeficiency virus.

CD4 - CD4 T-lymphocyte count in cells/μL.

### SARS-CoV-2 Sequencing of Upper Respiratory Specimens on Enrollment

Eight different SARS-CoV-2 phylogenetic lineages were detected from enrollment NP/OP swabs from the 10 randomly selected participants demonstrating high SARS-CoV-2 viral load shedding for > 2 weeks. These included 2 participants with B.1, 2 B.1.140, and 1 each of B.1.1.57, B.1.1.448, B.1.1.382, C.1, C.2, and C.6 lineages.

## DISCUSSION

In this South African cohort of 257 persons (including 102 PLHIV) hospitalized with COVID-19, the median duration of SARS-CoV-2 shedding was 13 days and did not differ significantly by HIV status. Among a subset with high initial SARS-CoV-2 viral shedding, duration of high viral load SARS-CoV-2 shedding (at N gene Ct value < 30) was 8 days overall and significantly prolonged in PLHIV with HIV immunosuppression and/or high HIV viral load. SARS-CoV-2 nucleic acids were detected in blood and stool/rectal swab specimens at low RNA concentrations in both PLHIV and HIV-uninfected persons. Immunocompromised PLHIV were also less likely to develop anti-spike protein antibody by day 7 or 14 compared to HIV-uninfected persons. A wide variety of SARS-CoV-2 phylogenetic lineages were detected in the small group of participants who shed SARS-CoV-2 at high viral loads for more than 2 weeks.

We showed prolonged duration of high viral load SARS-CoV-2 shedding in PLHIV with low CD4 cell counts. Prolonged influenza virus shedding has been described in immunocompromised PLHIV, and it has been associated with viral evolution and antigenic drift within individual immunocompromised patients, similar to that occurring in communities [31–34]. Further studies are needed to explore SARS-CoV-2 intra-individual adaptations in a similar manner to influenza, particularly in PLHIV.

The national prevalence of HIV infection in South Africa is 13%, and it is estimated that 4.4 million of the 7.5 million PLHIV in South Africa are not HIV virally suppressed. (UNAIDS https://www.unaids.org/en/regionscountries/countries/southafrica). If immunocompromised PLHIV shed SARS-CoV-2 for longer at higher SARS-CoV-2 viral loads, this could potentially impact the risk of viral evolution and transmission, especially in South Africa. Alternately, PLHIV with CD4 counts of ≥200 cells/µL shed for a similar time and at a similar SARS-CoV-2 viral load than HIV-uninfected persons. This finding highlights the importance of early HIV diagnosis and treatment with the goal of HIV viral suppression, thus improving the health of PLHIV and minimizing transmission risk of SARS-CoV-2 in the general population. The study was conducted prior to any COVID-19 vaccinations being available in South Africa. Now that vaccines are available, prioritizing COVID-19 vaccination of PLHIV may be another strategy for minimizing SARS-CoV-2 spread.

Recently, accelerated SARS-CoV-2 viral evolution was described in an immunocompromised host with persistent SARS-CoV-2 infection, as well as in a PLHIV host with poor HIV viral suppression [17, 35]. SARS-CoV-2 viral mutations occur commonly within hosts and within populations and prolonged high viral load shedding provides a good opportunity for virus evolution and transmission of potential viral mutations. Between June and October 2020, 44 different SARS-CoV-2 lineages were detected in South Africa, with the B.1, B.1.1, B.1.1.448, B.1.1.54, and C.1 lineages accounting for 59% of all sequences (n = 1705) [36]. In this study the multiple SARS-CoV-2 phylogenetic lineages detected reflect the lineage diversity during the earlier waves in South Africa, indicating that persistent shedding was not dominated by a specific lineage or variant. All lineages detected in these individuals had been identified in national genomic surveillance efforts, with 7 of the 8 being lineages first detected in South Africa [36]. Going forward we aim to investigate within host viral evolution by sequencing sequential specimens of the participants with prolonged high viral load shedding, many of whom were immunocompromised PLHIV.

Obese patients in our study showed a 45% increase in median time of SARS-CoV-2 shedding compared to their nonobese counterparts. However, obesity was not associated with prolonged duration of high viral load SARS-CoV-2 shedding. Recent studies indicate that obesity may be associated with prolonged SARS-CoV-2 shedding, in a similar manner to prolonged shedding seen in obese adults with influenza A virus [37, 38]. See Supplementary Material for further discussion.

Most participants developed anti-spike protein antibodies by day 14, with fewer immunocompromised PLHIV developing antibodies compared to HIV-uninfected persons. Overall, lower median anti-spike protein antibody titers were detected in PLHIV. Our study showed 14 individuals (4 HIV-uninfected persons and 10 PLHIV—8 of whom had low CD4 count) who did not develop antibodies within the initial 21 days’ follow-up period, half of whom showed prolonged shedding (including 5 PLHIV with CD4 count < 200 cells/µL). Other studies have found similar findings in immunocompromised individuals with prolonged viral shedding having negative seroconversion [15, 16].

The cohort of participants from 20 public hospitals is likely representative of the hospitalized COVID-19 South African adult population; however, the analysis did not account for within-hospital correlations due to case mix, treatment strategies, or interhospital differences in quality of care [39]. Thrice weekly collection of NP/OP swabs to detect ongoing SARS-CoV-2 viral shedding may result in underestimated viral shedding due to interval censoring of the data. A major limitation of our study is the use of qualitative Ct values as a proxy for viral load. Although all specimens were tested in the same laboratory, using standardized technique and assays, quantitative RT-PCR was not performed using calibration curves to determine viral load. In addition, we were unable to perform viral culture to confirm shedding of infectious virus at proxy Ct values < 30. Our findings may not be generalizable to outpatients with mild COVID-19, as hospitalized COVID-19 patients may represent more severe disease and therefore shed for a longer duration. Nonenrollment of participants due to unavailability of HIV test data (37% (702/1875) of nonenrollments) may have introduced bias due to potential differences in persons willing to undergo HIV testing versus those who are unwilling. Previous South African studies have shown similar unknown HIV results, as opt-out policies on HIV testing are not routinely followed [39]. Although sensitivity analyses in our study did not show much effect when excluding patients who died (data not shown), it should be noted that in-hospital mortality in our study was low (8%) compared to 23% in Jassat et al, as patients too ill to consent would have been excluded [39]. Follow-up of participants’ post-hospital discharge was challenging due to stigma of COVID-19 in the communities, safety concerns and travel limitations. Fifty-eight participants were lost to follow-up prior to cessation of shedding; however, the accelerated time failure Weibull regression analysis included patient data up until the patient was lost to follow-up, thereby utilizing all data generated.

Immunocompromised persons living with HIV shed SARS-CoV-2 at a higher viral load for a longer duration, and have lower anti-spike protein antibody levels than HIV-uninfected persons, which could lead to increased transmission and ongoing viral evolution of SARS-CoV-2, unless HIV control is achieved. During the COVID-19 pandemic, access to HIV testing and treatment with an aim for HIV viral suppression remains extremely important. In high HIV-prevalence settings better HIV control may potentially decrease the transmission period of SARS-CoV-2 in communities.

## Supplementary Data

Supplementary materials are available at *Clinical Infectious Diseases* online. Consisting of data provided by the authors to benefit the reader, the posted materials are not copyedited and are the sole responsibility of the authors, so questions or comments should be addressed to the corresponding author.

Supplementary Figure 1: Mosaic of relative SARS-CoV-2 C_t_-values of nasopharyngeal/oropharyngeal swabs taken every second day from persons living with HIV and HIV-uninfected persons who were hospitalized with COVID-19, South Africa, May through December 2020 (n = 257).

Supplementary Table 1: Reasons for non-enrolment by HIV status amongst persons screened for enrolment into the SARS-CoV-2 shedding study in South Africa

Supplementary Table 2: Accelerated Weibull Regression for duration of SARS-CoV-2 N gene detection by RT-PCR among hospitalized persons living with HIV, South Africa

Supplementary Table 3: Accelerated Weibull Regression for time taken from onset of symptoms to reach SARS-CoV-2 RT-PCR N gene C_t_-value of > 30 among a subset of hospitalized persons living with HIV with laboratory confirmed SARS-CoV-2 infection and initial study SARS-CoV-2 RT-PCR N gene C_t_-value of < 30, South Africa

Supplementary Table 4: Presence of SARS-CoV-2 virus in blood and stool among hospitalized persons with laboratory confirmed SARS-CoV-2 infection, South Africa

John Black: Department of Medicine, Walter Sisulu University, Port Elizabeth, Eastern Cape.

Pelonomi Academic and Universitas Academic Hospitals:

Dominique Goedhals: Division of Virology, National Health Laboratory Service (NHLS)/ University of the Free State, Bloemfontein, Free State.

Bonnie Maloba: Microbiology, University of the Free State, Bloemfontein Free State.

Samantha Potgieter: Internal Medicine, Division Infectious Diseases, University of the Free State, Bloemfontein, Free State.

Charlotte Maxeke Johannesburg Academic Hospital:

Marianne Black: Department of Clinical Microbiology and infectious Diseases, University of the Witwatersrand, Johannesburg, Gauteng Microbiology, NHLS, Johannesburg, Gauteng.

Vindana Chibabhai: Clinical Microbiology, NHLS, Johannesburg, Gauteng Clinical Microbiology and Infectious Diseases, University of the Witwatersrand, Johannesburg, Gauteng.

Nonhlanhla Mbenenge: Virology, NHLS, Johannesburg, Gauteng Virology, University of the Witwatersrand, Johannesburg Gauteng.

Trusha Nana: Microbiology, NHLS, Johannesburg, Gauteng Department of Clinical Microbiology and Infectious Diseases, University of Witwatersrand, Johannesburg, Gauteng.

Sarah Stacey: Department of Internal Medicine, University of the Witwatersrand, Johannesburg, Gauteng Department of Internal Medicine, Charlotte Maxeke Johannesburg Academic Hospital, Johannesburg, Gauteng.

Florette Treurnicht: Virology, NHLS, Johannesburg, Gauteng.

Dr George Mukhari Academic Hospital.

Masego Moncho: Microbiology, NHLS, Pretoria, Gauteng Microbiology, Sefako Makgatho Health Sciences University, Pretoria, Gauteng.

Maphoshane Nchabeleng: Microbiology, NHLS, Pretoria, Gauteng Microbiology, Sefako Makgatho Health Sciences University, Pretoria, Gauteng.

Grace Shikwambane-Ntlemo: Microbiology, NHLS, Pretoria, Gauteng Microbiology, Sefako Makgatho Health Sciences University, Pretoria, Gauteng Helen Joseph Hospital.

Rispah Chomba: Clinical microbiology, NHLS, Johannesburg, Gauteng Department of clinical microbiology and infectious diseases, School of Pathology, University of the Witwatersrand, Johannesburg, Gauteng.

Jeremy Nel: Division of Infectious Diseases, Department of Medicine, University of the Witwatersrand, Johannesburg, Gauteng.

Steve Biko Academic Hospital.

Anwar Hoosen: Medical Microbiology, University of Pretoria, Pretoria, Gauteng Microbiology Laboratory, NHLS, Tshwane Academic Division, Pretoria, Gauteng.

Mohamed Said: Medical Microbiology, University of Pretoria, Pretoria, Gauteng Tshwane Academic Division, NHLS, Pretoria, Gauteng.

Addington Hospital.

Junaid Bayat: Medicine, Addington Hospital, Durban, KwaZulu Natal.

Lisha Sookan: Medical Microbiology, NHLS, Durban, KwaZulu Natal Medical Microbiology, University of KwaZulu Natal, Durban, KwaZulu Natal.

Khine Swe Han: Medical Microbiology, NHLS, Durban, KwaZulu Natal

Edendale and Northdale Hospitals.

Surendra Sirkar: Department of Health, Northdale Hospital, Pietermaritzburg, KwaZulu Natal

Greys Hospital.

Halima Dawood: Medicine, Greys hospital, Pietermaritzburg, KwaZulu Natal Caprisa, University of Kwazulu Natal, Durban, KwaZulu Natal.

Sumayya Haffejee: Medical Microbiology, NHLS, Pietermaritzburg, KwaZulu Natal Medical Microbiology, University of KwaZulu-Natal, Pietermaritzburg, KwaZulu Natal.

King Edward III Hospital.

Somasundram Pillay: Internal Medicine, University of KwaZulu-Natal, Durban, KwaZulu Natal Internal Medicine, King Edward VIII Hospital, Durban, KwaZulu Natal.

Praksha Ramjathan: Medical Microbiology, NHLS, Durban, KwaZulu Natal Medical Microbiology, University of KwaZulu Natal, Durban, KwaZulu Natal.

RK Khan Hospital.

Nomonde Mvelase: Medical Microbiology, NHLS, Durban, KwaZulu Natal Medical Microbiology, University of KwaZulu-Natal, Durban, KwaZulu Natal.

Javid Mulla: Medicine, RK Khan and King Edward III Hospitals, Durban, KwaZulu Natal

Polokwane Provincial and Mankweng Hospitals.

Ruth Lekalakala-Mokaba: Pathology, University of Limpopo, Polokwane, Limpopo Medical Microbiology, NHLS, Polokwane, Limpopo.

Rob Ferreira Hospital.

Matamela Madua: Medicine, Rob Ferreira Hospital, Nelspruit, Mpumalanga.

Sindile Ntuli: Medical Microbiology, NHLS, Nelspruit, Mpumalanga Tshepong Hospital

Ebrahim Variava: Medicine, Klerksdorp/Tshepong Hospital Complex, Klerksdorp, North West

Mitchells Plain Hospital.

Thomas Crede: Medicine, Mitchells Plain Hospital, Cape Town, Western Cape Medicine, University of Cape Town, Cape Town, Western Cape.

Tygerberg Hospital.

Kessendri Reddy: Division of Medical Microbiology and Immunology, Department of Pathology, Stellenbosch University, Cape Town, Western Cape Medical Microbiology, NHLS, Tygerberg Hospital, Cape Town, Western Cape.

Jantjie Taljaard: Division of Infectious Diseases, Department of Medicine, Tygerberg Hospital and Stellenbosch University, Cape Town, Western Cape.

Andrew Whitelaw: Division of Medical Microbiology and Immunology, Department of Pathology, Stellenbosch University, Cape Town, Western Cape Medical Microbiology, NHLS, Tygerberg Hospital, Cape Town, Western Cape.

ciac077_suppl_Supplementary_Appendix

ciac077_suppl_Supplementary_Figure_S1

ciac077_suppl_Supplementary_Legends

## Notes

***Acknowledgments.*** The authors thank the participants in the study for their time and patience, as well as the GERMS-SA surveillance officers and team members of the Centre for Respiratory Diseases and Meningitis at the NICD.

Although not part of the COVID-19 Shedding Study Group, the following individuals assisted in the study:

Livingstone Hospital: Vanessa Pearce, Microbiology, National Health Laboratory Service, Eastern Cape; Pelonomi Academic Hospital: Dewald Steyn, Internal Medicine, Pelonomi Hospital, Bloemfontein, Free State; Dr George Mukhari Academic Hospital: Temitayo Famoroti, Clinical Virology, National Health Laboratory Service, Gauteng; and Nkosinathi Mdladla, Medicine, Dr George Mukhari Hospital, Gauteng; King Edward III Hospital: Prasha Mahabeer, Microbiology, National Health Laboratory Service, Kwazulu-Natal; Mankweng Hospital: Samuel Cana, Medicine, Mankweng Hospital, Limpopo.

Members of the COVID-19 Shedding Study Group and their affiliations.

Members from the various hospital sites that made up the COVID-19 Shedding Study Group.

Dora Nginza Provincial and Livingstone Hospitals:

***Author contributions.*** Conception and design of study: All authors.

Data collection and laboratory processing: S. M., S. T., J. N. B., A. B., J. K., M. M., M. McM., J. M., V. Q., S, W. , M. d. P., NW, A. v. G., C. C.

Analysis and interpretation: S. M., S. T., C. C., A. v. G., J. N. B., A. B., J. K., M. McM., J. M., V. Q., S, W.

Drafting or critical review of the article: All authors.

***Financial support.*** The study was funded by the Wellcome Trust (grant number 221003/Z/20/Z) in collaboration with the Foreign, Commonwealth and Development Office, United Kingdom; the US Centers for Disease Control and Prevention (co-operative agreement number: 6 U01IP001048-04-02), as well as the National Institute for Communicable Diseases, a division of the National Health Laboratory Service, South Africa. The funding agencies had no role in the development of the study protocol, data collection, analysis and interpretation, writing of the report, or decision to submit.

***Potential conflicts of interest.*** S. M. reports a grant from Sanofi Pasteur for research outside the submitted work. A. v. G. reports grants from US CDC Atlanta and CDC SA, Wellcome Trust, Sanofi, and from South African MRC, EDCTP partnership, BMFG through ASLM and Africa CDC, Fleming Fund Regional Africa WGS (SEQAFRICA) outside the submitted work. C. C. reports grants from Wellcome Trust, SA Medical Research Council (SAMRC), and US CDC related to the summited work and grant to institute for COVID-19 Research from Sanofi Pasteur outside the submitted work; participation on a Member Scientific Advisory Committee—BCHW: Burden of COVID-19 among health care workers, assessing infection, risk factors, vaccine effectiveness, working experiences and one-health implications: a mixed methodology, multisite international study, study is focused on COVID-19—no tuberculosis. H. D. has received speaker fees from SANOFI, unrelated to the study. All other authors report no potential conflicts. All authors have submitted the ICMJE Form for Disclosure of Potential Conflicts of Interest. Conflicts that the editors consider relevant to the content of the manuscript have been disclosed.
