# Supplementary material for: Prolonged Shedding of Severe acute respiratory syndrome coronavirus 2 (SARS-CoV-2) at High Viral Loads Among Hospitalized Immunocompromised Persons Living With Human Immunodeficiency Virus (HIV), South Africa
Source: Clin Infect Dis. 2022 Feb 2;75(1):e144–56. doi: 10.1093/cid/ciac077 (PMC8903337; doi:10.1093/cid/ciac077)
Supplement: ciac077_suppl_Supplementary_Appendix [file ciac077_suppl_supplementary_appendix.docx]

SUPPLEMENTARY APPENDIX

TITLE:

Prolonged shedding of SARS-CoV-2 at high viral loads amongst hospitalised immunocompromised persons living with HIV, South Africa

*This appendix is designed to be read in conjunction with the full manuscript in Clinical Infectious Diseases journal which refers to the various sections below.*

SUPPLEMENTARY METHODS

*Study design*

Using standardised case report forms, demographic and clinical details were collected at enrolment, daily whilst in hospital, and at discharge from hospital/cessation of shedding/death of the participant. Clinical details included: age, sex, race, presence of comorbidities (diabetes, hypertension, chronic lung/renal/cardiac disease, tuberculosis on treatment, obesity (body mass index >30), smoking history, signs and symptoms of SARS-CoV-2 infection, WHO classification of COVID-19 severity, length of hospital stay, ventilation requirements and in-hospital treatment.^1^ For PLHIV, CD4 T-lymphocyte count and HIV-viral load within 3 months of hospital admission and current use of antiretroviral therapy were documented. A specific variable was created indicating HIV immunosuppression incorporating categories for HIV-uninfected persons, PLHIV with CD4 count >200cells/µl, PLHIV with CD4 count <200cells/µl and PLHIV without a documented CD4 count. A similar variable was created indicating HIV viral suppression including the following categories: HIV-uninfected, PLHIV with HIV viral load <400copies/ml, PLHIV with HIV viral load >400copies/ml and PLHIV without a documented HIV viral load.

*Laboratory diagnostics*

Total nucleic acids were extracted from specimens using a MagNA Pure 96 automated extractor and DNA/Viral NA Small Volume v2.0 extraction kit (Roche Diagnostics, Mannheim, Germany). Real-time reverse transcription polymerase chain reaction (rRT-PCR) for the qualitative detection of nucleic acid from SARS-CoV-2 was performed on NP/OP, stool/rectal swabs and blood specimens using the Allplex™ nCoV 2019 kit (Seegene, Seoul, South Korea). The assay detects three gene targets of the SARS-CoV-2 virus (envelope (E), RNA-dependent RNA-polymerase (RdRP) and nucleocapsid (N gene)). Specimens were considered positive for SARS-CoV-2 nucleic acids if the C_t_ was <40 for ≥1 gene target. An N gene C_t_-value <30 on NP/OP specimens was used as a proxy for a high viral load based on published data showing a high correlation between low C_t_-values (using various gene targets), high viral load and increased odds of shedding cultivable virus.^2–5^

SARS-CoV-2 sequencing was performed on the first NP/OP specimens on enrolment from ten randomly selected participants of 29, who demonstrated high viral load shedding for >14 days. We used AmpliSeq for SARS-CoV-2 (Illumina), on the Ion Torrent Genexus platform. Genomes were assembled using the Exatype SARS-CoV-2 pipeline (https://sars-cov-2.exatype.com/). Clade and lineage assignments were made using the online Nextclade (https://clades.nextstrain.org/) and Pangolin (https://pangolin.cog-uk.io/) applications, which also enable identification of known variants of concern as well as novel mutations.

SUPPLEMENTARY RESULTS

*SARS-CoV-2 shedding in rectal swabs/stool and blood*

Amongst all participants with available specimens, 18% (31/171) were SARS-CoV-2 rRT-PCR positive on blood and 34% (68/201) were positive on rectal swab/stool, within the first 7 days post-symptom onset. At 14 days, 18% (9/51) were positive on blood and 27% (43/157) on rectal swab/stool, with no difference by HIV status (p=0.22 blood, and p=0.65 rectal swab/stool). After 21 days 10% (3/31) were positive on blood and 21% (22/103) on rectal swab/stool. All median SARS-CoV-2 N gene C_t_-values for both blood and rectal swabs/stool at any time point sampled were above 35 (Supplementary table 4).

SUPPLEMENTARY DISCUSSION

Obese patients in our study showed a 45% increase in median time of SARS-CoV-2 shedding (18 days), compared to the 13 days in their non-obese counterparts. However, we did not show obesity to be associated with prolonged duration of high viral load SARS-CoV-2 shedding.

Obese persons are at risk of severe disease and mortality from COVID-19, possibly due to high leptin levels, high levels of chemokines, and a compromised immune response that fails to contain viral replication.^6,7^ Recent studies indicate that obesity may be associated with prolonged SARS-CoV-2 shedding, in a similar manner to prolonged shedding seen in obese adults with influenza A virus. ^8,9^ Increased adipose tissue (containing increased angiotensin converting enzyme II receptors) may also serve as a reservoir for viral persistence, which could be the source of continuous viral shedding.^10^

LIST OF FIGURES AND TABLES

Supplementary figure 1: Mosaic of relative SARS-CoV-2 C_t_-values of nasopharyngeal/oropharyngeal swabs taken every second day from persons living with HIV and HIV-uninfected persons who were hospitalised with COVID-19, South Africa, May through December 2020.

Supplementary table 1: Reasons for non-enrolment by HIV status amongst persons screened for enrolment into the SARS-CoV-2 shedding study in South Africa

Supplementary table 2: Accelerated Weibull Regression for duration of SARS-CoV-2 N gene detection by RT-PCR among hospitalised persons living with HIV, South Africa

Supplementary table 3: Accelerated Weibull Regression for time taken from onset of symptoms to reach SARS-CoV-2 RT-PCR N gene C_t_-value of >30 among a subset of hospitalised persons living with HIV with laboratory confirmed SARS-CoV-2 and initial study SARS-CoV-2 RT-PCR N gene C_t_-value of <30, South Africa

Supplementary table 4: Presence of SARS-CoV-2 virus in blood and stool among hospitalised persons with laboratory confirmed SARS-CoV-2, South Africa

REFERENCES

1 WHO. Clinical management Clinical management Living guidance COVID-19. World Heal. Organ. 2021. https://www.who.int/publications/i/item/WHO-2019-nCoV-clinical-2021-1.

2 Singanayagam A, Patel M, Charlett A, *et al.* Duration of infectiousness and correlation with RT-PCR cycle threshold values in cases of COVID-19, England, January to May 2020. *Eurosurveillance* 2020; **25**. DOI:10.2807/1560-7917.ES.2020.25.32.2001483.

3 Public Health Ontario, Group TOC-19 TTW. Focus on: an overview of cycle threshold values and their role in SARS-CoV-2 real-time PCR test interpretation. Queen’s Print. Ontario. 2020; : 1–14.

4 Bullard J, Dust K, Funk D, *et al.* Predicting Infectious Severe Acute Respiratory Syndrome Coronavirus 2 From Diagnostic Samples. *Clin Infect Dis* 2020; published online May 22. DOI:10.1093/cid/ciaa638.

5 Tom MR, Mina MJ. To Interpret the SARS-CoV-2 Test, Consider the Cycle Threshold Value. *Clin Infect Dis* 2020; **02115**: 1–3.

6 Richter FC, Alrubayyi A, Crespo AT, Hulin-Curtis S. Impact of obesity on host defences: Implications for SARS-CoV-2 infection. *Oxford Open Immunol* 2021; **0**: 1–9.

7 Rebello CJ, Kirwan JP, Greenway FL. Obesity, the most common comorbidity in SARS-CoV-2: is leptin the link? *Int J Obes* 2020; **44**: 1810–7.

8 Moriconi D, Masi S, Rebelos E, *et al.* Obesity prolongs the hospital stay in patients affected by COVID-19, and may impact on SARS-COV-2 shedding. *Obes Res Clin Pract* 2020; **14**: 205–9.

9 Maier HE, Lopez R, Sanchez N, *et al.* Obesity Increases the Duration of Influenza A Virus Shedding in Adults. *J Infect Dis*; **1378**: 218.

10 Bhattacharya I, Ghayor C, Pérez Dominguez A, Weber FE. From Influenza Virus to Novel Corona Virus (SARS-CoV-2)–The Contribution of Obesity. *Front Endocrinol (Lausanne)* 2020; **11**. DOI:10.3389/fendo.2020.556962.

Supplementary figure 1: Mosaic of relative SARS-CoV-2 C_t_-values of nasopharyngeal/oropharyngeal swabs taken every second day from persons living with HIV and HIV-uninfected persons who were hospitalised with COVID-19, South Africa, May through December 2020 (n=257)


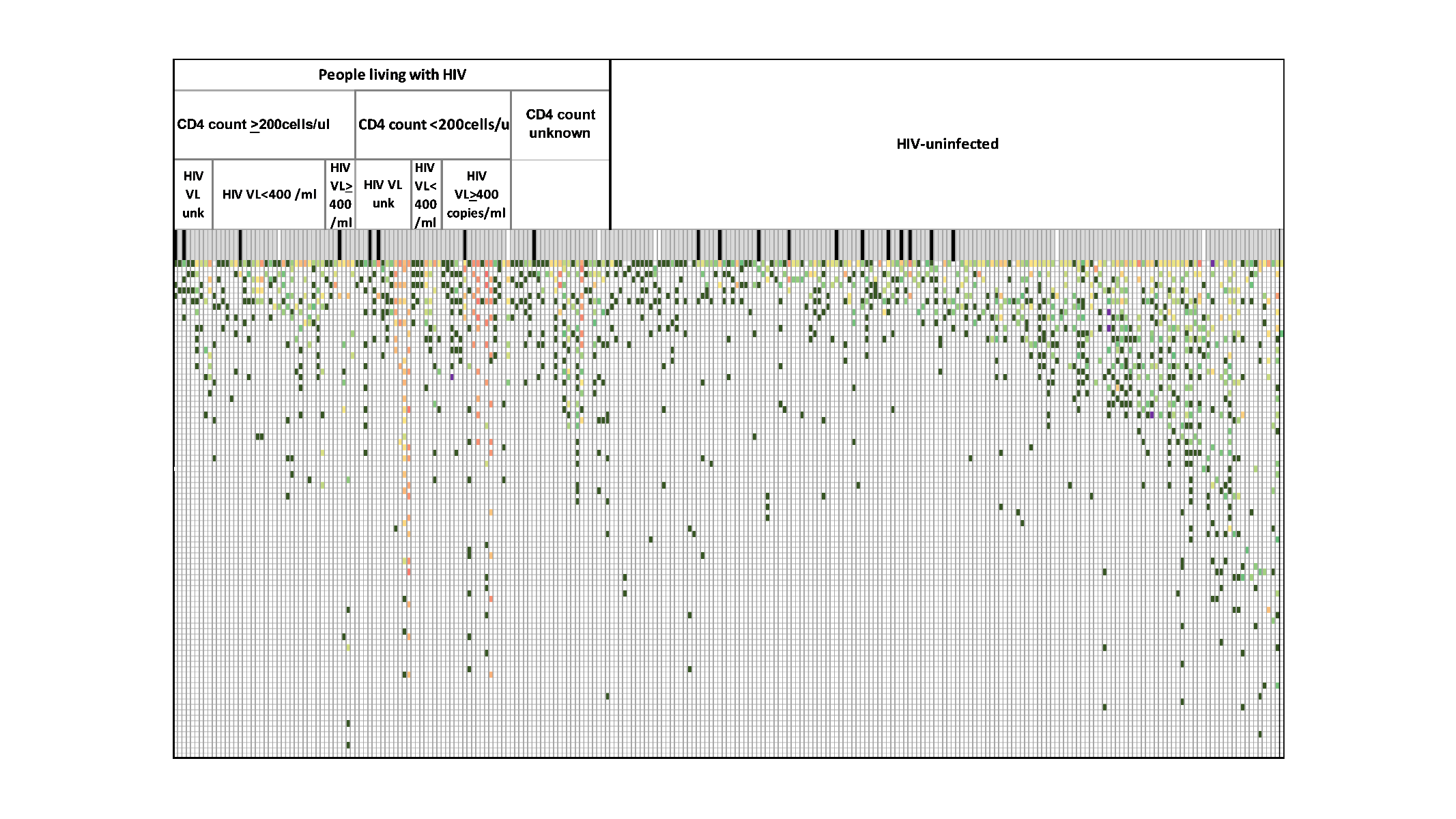


Footnote: Each vertical column denotes one patient. The black rectangles denote patients who died. Each coloured block denotes results of rRT-PCR for SARS-CoV-2 N gene on combined nasopharyngeal/oropharyngeal swab taken at various time points from day 1 of enrolment. C_t_-values of SARS-CoV-2 swabs are indicated on a colour scale from lowest C_t_ value in orange to mid-values (24-29) in yellows to higher values (30-39) in light green to dark green indicating C_t_-value>40/not detected).

Supplementary table 1: Reasons for non-enrolment by HIV status amongst persons screened for enrolment into the SARS-CoV-2 shedding study in South Africa

|  | **HIV-uninfected** | | **HIV-infected** | | **HIV unknown** | | **Total** |
| --- | --- | --- | --- | --- | --- | --- | --- |
| **Reasons for non-enrollment** | **n** | **%** | **n** | **%** | **n** | **%** | **N** |
| Refused consent | 122 | 54% | 46 | 20% | 57 | 25% | **225** |
| Transferred to another hospital before admission | 105 | 56% | 34 | 18% | 47 | 25% | **186** |
| Less than 18 years of age | 14 | 22% | 8 | 13% | 42 | 66% | **64** |
| Hospitalised for non-medical reasons (isolation only) | 113 | 43% | 44 | 17% | 105 | 40% | **262** |
| Lived >50km radius from hospital | 56 | 50% | 22 | 20% | 33 | 30% | **111** |
| Screened >5 days post initial COVID-19 test | 125 | 38% | 67 | 21% | 133 | 41% | **325** |
| No HIV result available on admission |  |  |  |  | 702 | 100% | **702** |
|  | **535** |  | **221** |  | **1119** |  | **1875** |

**Supplementary Table 2: Accelerated Weibull Regression for duration of SARS-CoV-2 N gene detection by RT-PCR among hospitalised persons living with HIV, South Africa**

| **Characteristic** | **Number included in each category in survival analysis** | **Duration of shedding (median) in days** | **Univariate analysis** | | | **Multivariable analysis** | | |
| --- | --- | --- | --- | --- | --- | --- | --- | --- |
|  |  | **mean (IQR^a^)** | **Hazard ratio** | **95% Confidence interval** | **p-value** | **Hazard ratio** | **95% Confidence interval** | **p-value** |
| **Age category (years) (n=100)** | |  |  |  |  |  |  |  |
| 18-34 years | 28 | 9 (1-37) | reference |  |  |  |  |  |
| 35-59 years | 62 | 12 (7-19) | 1 | 0.59-1.72 | 0.972 |  |  |  |
| 60+ years | 10 | 7 (7-34) | 0.89 | 0.34-2.38 | 0.823 |  |  |  |
| **Sex (n=101)** |  |  |  |  |  |  |  |  |
| Male | 24 | 10 (4-16) | 0.99 | 0.54-1.83 | 0.987 |  |  |  |
| Female | 77 | 11 (5-27) |  |  |  |  |  |  |
| **CD4^b^ count (n=78)** |  |  |  |  |  |  |  |  |
| <200 cells/µl | 37 | 14 (4-29) | reference |  |  |  |  |  |
| >200 cells/µl | 41 | 11 (7-17) | 1.22 | 0.69-2.15 | 0.499 |  |  |  |
| **HIV Viral suppression (n=63)** | |  |  |  |  |  |  |  |
| <400 copies/ml | 37 | 13 (6-17) | reference |  |  |  |  |  |
| >400 copies/ml | 26 | 14 (4-37) | 0.54 | 0.28-1.06 | 0.072 | 0.36 | 0.13-0.98 | 0.046 |
| **ARV use (n=80)** |  |  |  |  |  |  |  |  |
| Yes | 67 | 10 (4-22) | 1.87 | 0.84-4.15 | 0.127 | 7.93 | 0.77-81.96 | 0.082 |
| No | 13 | 13 (6-37) |  |  |  |  |  |  |
| **Glucocorticoid use (n=95)** |  |  |  |  |  |  |  |  |
| Yes | 33 | 16 (6-27) | 1.04 | 0.80-4.22 | 0.591 |  |  |  |
| No | 62 | 11 (4-17) | reference |  |  |  |  |  |
| **Severity of illness (n=92)** |  |  |  |  |  |  |  |  |
| Mild | 19 | 15 (4-19) | reference |  |  |  |  |  |
| Moderate | 60 | 10 (6-17) | 1.25 | 0.66-2.38 | 0.49 |  |  |  |
| Severe | 13 | 20 (0-37) | 0.46 | 0.16-1.31 | 0.145 |  |  |  |
| **Specific comorbidities** |  |  |  |  |  |  |  |  |
| Hypertension (n=98) | 24 | 10 (7-16) | 1.34 | 0.76-2.37 | 0.313 |  |  |  |
| no hypertension | 74 | 13 (5-25) |  |  |  |  |  |  |
| Diabetes (n=98) | 11 | 13 (8-17) | 0.54 | 0.20-1.49 | 0.233 |  |  |  |
| no diabetes | 87 | 11 (5-27) | reference |  |  |  |  |  |
| Obesity (n=98) | 13 | 19 (13-36) | 0.56 | 0.28-1.14 | 0.113 | 0.26 | 0.06-1.14 | 0.073 |
| no obesity | 85 | 10 (5-20) | reference |  |  |  |  |  |
| Tuberculosis (n=98) | 16 | 14 (4-28) | 1.16 | 0.63-2.13 | 0.637 |  |  |  |
| no TB | 82 | 11 (6-22) | reference |  |  |  |  |  |
| **Smoking history (n=75)** |  |  |  |  |  |  |  |  |
| Current smoker | 9 | 4 (1-10) | reference |  |  |  |  |  |
| Former smoker | 9 | 17 (13-22) | 0.37 | 0.12-1.15 | 0.086 | 0.04 | 0.002-0.81 | 0.036 |
| Non-smoker | 57 | 11 (6-30) | 0.41 | 0.17-0.99 | 0.048 | 0.06 | 0.01-0.56 | 0.013 |

Footnote: ^a^IQR – interquartile range; ^b^CD4 – CD4 T lymphocyte count in cells/µl

**Supplementary Table 3: Accelerated Weibull Regression for time taken from onset of symptoms to reach SARS-CoV-2 RT-PCR N gene C_t_ -value of >30 among a subset of hospitalised persons living with HIV with laboratory confirmed SARS-CoV-2 infection and initial study SARS-CoV-2 RT-PCR N gene C_t_-value of <30, South Africa**

| **Characteristic** | **Number included in each category in survival analysis** | **Time from symptom onset to reach SARS-CoV-2 N gene C_t_ value>30** | **Univariate analysis** | | | **Multivariable analysis** | | |
| --- | --- | --- | --- | --- | --- | --- | --- | --- |
|  |  | **mean (IQR^a^)** | **Hazard ratio** | **95% Confidence interval** | **p-value** | **Hazard ratio** | **95% Confidence interval** | **p-value** |
| **Age category (years) (n=40)** |  |  |  |  |  |  |  |  |
| 18-34 years | 11 | 13 (3-50) | reference |  |  |  |  |  |
| 35-59 years | 25 | 10 (4-26) | 1.33 | 0.62-2.85 | 0.462 |  |  |  |
| 60+ years | 4 | 7 (6-17) | 1.82 | 0.48-6.83 | 0.375 |  |  |  |
| **Sex (n=41)** |  |  |  |  |  |  |  |  |
| Male | 13 | 8 (4-20) | 0.94 | 0.44-2.01 | 0.873 |  |  |  |
| Female | 28 | 10 (5-34) | reference |  |  |  |  |  |
| **CD4^b^ count (n=32)** |  |  |  |  |  |  |  |  |
| <200 cells/µl | 18 | 27 (8-43) | reference |  |  |  |  |  |
| >200 cells/µl | 14 | 7 (4-10) | 7.25 | 2.80-18.79 | <0.001 |  |  |  |
| **HIV Viral suppression (n=24)** |  |  |  |  |  |  |  |  |
| <400 copies/ml | 11 | 6 (4-8) | reference |  |  |  |  |  |
| >400 copies/ml | 13 | 26 (10-41) | 0.14 | 0.05-0.43 | 0.001 | 0.05 | 0.01-0.29 | 0.001 |
| **CD4 and HIV viral load category (n=23)** |  |  |  |  |  |  |  |  |
| CD4 <200 and VL <400 | 2 | 5 (3-8) | reference |  |  |  |  |  |
| CD4<200 and VL >400 | 9 | 37 (26-43) | 0.03 | 0.01-0.23 | 0.001 |  |  |  |
| CD4>200 and VL >400 | 4 | 9 (5-10) | 0.53 | 0.10-2.92 | 0.468 |  |  |  |
| CD4>200 and VL <400 | 8 | 5 (4-7) | 0.55 | 0.11-2.70 | 0.463 |  |  |  |
| **ARVuse (n=32)** |  |  |  |  |  |  |  |  |
| Yes | 25 | 7 (3-22) | 2.01 | 0.80-5.00 | 0.135 | 0.76 | 0.21-2.74 | 0.674 |
| No | 7 | 37 (8-50) | reference |  |  |  |  |  |
| **Glucocorticoid use (n=37)** | |  |  |  |  |  |  |  |
| Yes | 13 | 26 (19-37) | 0.73 | 0.36-1.49 | 0.392 | 1.35 | 0.37-4.90 | 0.652 |
| No | 24 | 8 (4-15) | reference |  |  |  |  |  |
| **Severity of illness (n=38)** |  |  |  |  |  |  |  |  |
| Mild | 8 | 10 (7-31) | reference |  |  |  |  |  |
| Moderate | 23 | 8 (4-26) | 1.33 | 0.53-3.31 | 0.539 |  |  |  |
| Severe | 7 | 37 (10-43) | 0.84 | 0.27-2.62 | 0.769 |  |  |  |
| **Specific comorbidities** |  |  |  |  |  |  |  |  |
| Hypertension (n=40) | 7 | 10 (8-26) | 1.03 | 0.42-2.48 | 0.956 |  |  |  |
| Diabetes (n=40) | 4 | 7 (3-43) | 0.22 | 0.03-1.63 | 0.139 |  |  |  |
| Obesity (n=40) | 6 | 18 (8-43) | 1.01 | 0.42-2.44 | 0.981 |  |  |  |
| Tuberculosis (n=40) | 8 | 23 (6-40) | 0.98 | 0.44-2.17 | 0.964 |  |  |  |
| **Smoking history (n=30)** |  |  |  |  |  |  |  |  |
| Current smoker | 4 | 7 (1-25) | reference |  |  |  |  |  |
| Former smoker | 3 | 20 (4-62) | 0.62 | 0.12-3.10 | 0.562 |  |  |  |
| Non-smoker | 23 | 8 (5-43) | 0.65 | 0.19-2.21 | 0.490 |  |  |  |
| **Spike ODB >0.4 by day 7 post symptom onset (n=30)** | | |  |  |  |  |  |  |
| No | 11 | 10 (7-26) | reference |  |  |  |  |  |
| Yes | 19 | 5 (2-8) | 1.95 | 0.84-4.54 | 0.119 |  |  |  |
| **Spike ODB >0.4 at day 14 post symptom onset (n=25)** | | | |  |  |  |  |  |
| No | 8 | 40 (21-70) | reference |  |  |  |  |  |
| Yes | 17 | 5 (2-13) | 6.58 | 2.32-18.65 | <0.001 |  |  |  |

Footnote: ^a^IQR – interquartile range;  ^b^CD4 – CD4 T lymphocyte count in cells/µl

Supplementary table 4: Presence of SARS-CoV-2 virus in blood and stool among hospitalised persons with laboratory confirmed SARS-CoV-2, South Africa

|  | **All (n= 257)** | | **HIV negative (n=155 )** | | **HIV positive (n= 102)** | | **Univariate analysis** | | |
| --- | --- | --- | --- | --- | --- | --- | --- | --- | --- |
|  | **Median or n/N** | **Inter quartile range or Percent** | **Median or n/N** | **Inter quartile range or Percent** | **Median or n/N** | **Inter quartile range or Percent** | **odds ratio** | **95% confidence interval** | **p-value** |
| **Median N gene** C_t_ **value on blood samples by day 7** | 37.47 | 36.06-38.49 | 37.55 | 36.29-38.49 | 36.6 | 35.07-38.62 |  |  | 0.685 |
| **SARS-CoV-2 PCR positive on blood samples by day 7 post symptom onset** | 31/171 | 18 | 23/108 | 21 | 8/63 | 13 | 0.54 | 0.22-1.29 | 0.163 |
| **SARS-CoV-2 PCR positive on blood samples at day 14 post symptom onset** | 9/51 | 18 | 4/32 | 13 | 5/19 | 26 | 2.5 | 0.58-10.8 | 0.22 |
| **SARS-CoV-2 PCR positive on blood samples at day 21 post symptom onset** | 3/31 | 10 | 1/20 | 5 | 2/11 | 18 | 4.22 | 0.34-52.90 | 0.264 |
|  |  |  |  |  |  |  |  |  |  |
| **Median N gene** C_t_ **value on stool samples by day 7** | 35.33 | 32.88-37.54 | 35.35 | 32.73-37.48 | 35.33 | 33.06-37.93 |  |  | 0.8301 |
| **SARS-CoV-2 PCR positive on stool samples by day 7 post symptom onset** | 68/201 | 34 | 42/121 | 35 | 26/80 | 33 | 0.91 | 0.5-1.65 | 0.746 |
| **SARS-CoV-2 PCR positive on stool samples at day 14 post symptom onset** | 43/157 | 27 | 27/94 | 29 | 16/63 | 25 | 0.84 | 0.41-1.74 | 0.647 |
| **SARS-CoV-2 PCR positive on stool samples at day 21 post symptom onset** | 22/103 | 21 | 14/59 | 24 | 8/44 | 18 | 0.71 | 0.27-1.89 | 0.498 |
