# Supplementary figures and images for: Prolonged Shedding of Severe acute respiratory syndrome coronavirus 2 (SARS-CoV-2) at High Viral Loads Among Hospitalized Immunocompromised Persons Living With Human Immunodeficiency Virus (HIV), South Africa
Source: Clin Infect Dis. 2022 Feb 2;75(1):e144–56. doi: 10.1093/cid/ciac077 (PMC8903337; doi:10.1093/cid/ciac077)

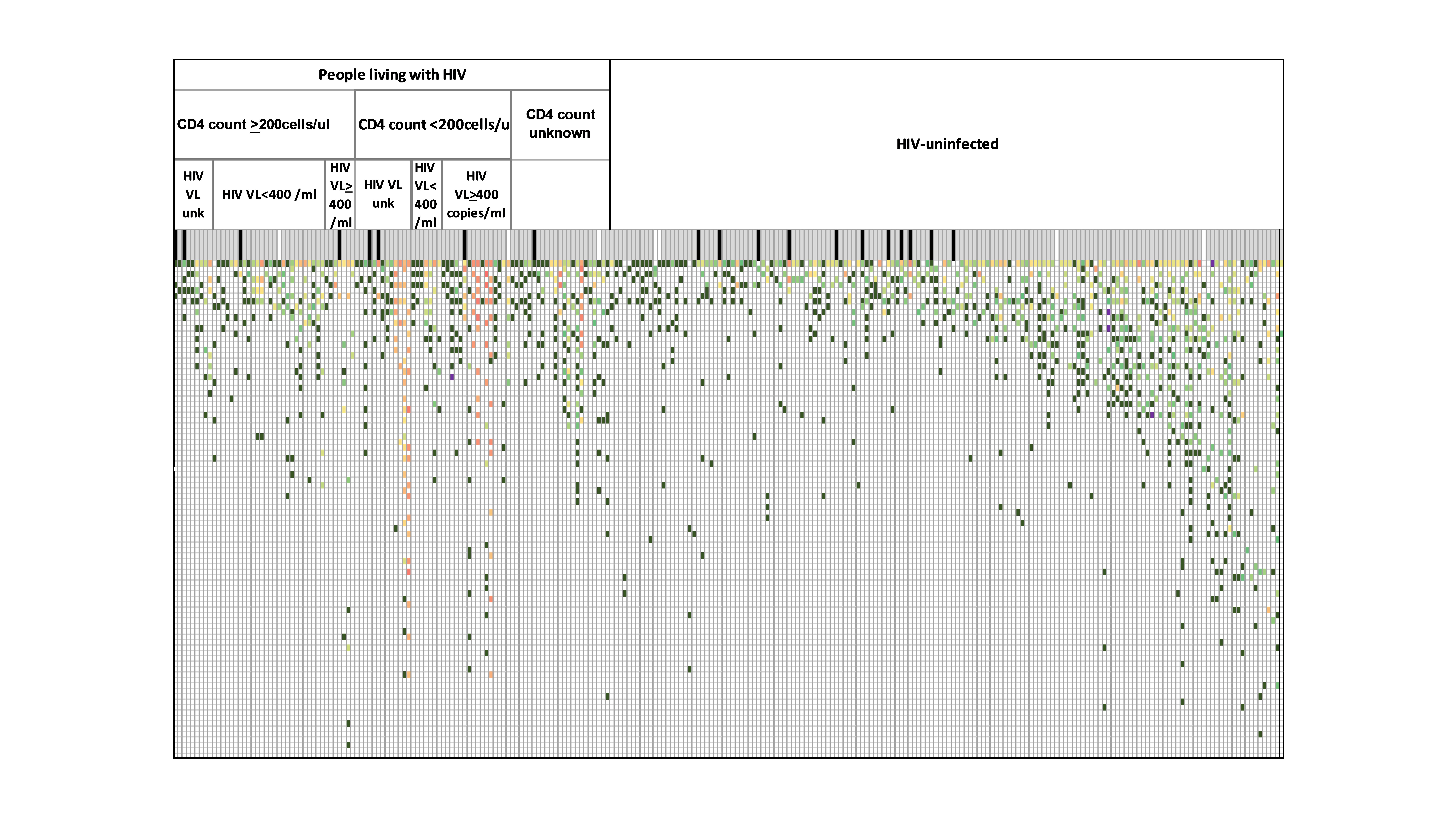

Supplement: ciac077_suppl_Supplementary_Figure_S1 [file ciac077_suppl_supplementary_figure_s1.jpeg]
