# Supplementary material for: Prolonged Shedding of Severe acute respiratory syndrome coronavirus 2 (SARS-CoV-2) at High Viral Loads Among Hospitalized Immunocompromised Persons Living With Human Immunodeficiency Virus (HIV), South Africa
Source: Clin Infect Dis. 2022 Feb 2;75(1):e144–56. doi: 10.1093/cid/ciac077 (PMC8903337; doi:10.1093/cid/ciac077)
Supplement: ciac077_suppl_Supplementary_Legends [file ciac077_suppl_supplementary_legends.docx]

Supplementary figure 1: Mosaic of relative SARS-CoV-2 C_t_-values of nasopharyngeal/oropharyngeal swabs taken every second day from persons living with HIV and HIV-uninfected persons who were hospitalised with COVID-19, South Africa, May through December 2020 (n=257)

Supplementary table 1: Reasons for non-enrolment by HIV status amongst persons screened for enrolment into the SARS-CoV-2 shedding study in South Africa

Supplementary table 2: Accelerated Weibull Regression for duration of SARS-CoV-2 N gene detection by RT-PCR among hospitalised persons living with HIV, South Africa

Supplementary table 3: Accelerated Weibull Regression for time taken from onset of symptoms to reach SARS-CoV-2 RT-PCR N gene C_t_-value of >30 among a subset of hospitalised persons living with HIV with laboratory confirmed SARS-CoV-2 infection and initial study SARS-CoV-2 RT-PCR N gene C_t_-value of <30, South Africa

Supplementary table 4: Presence of SARS-CoV-2 virus in blood and stool among hospitalised persons with laboratory confirmed SARS-CoV-2 infection, South Africa
